# Biological, environmental, and psychological stress and the human gut microbiome in healthy adults

**DOI:** 10.1038/s41598-024-77473-9

**Published:** 2025-01-02

**Authors:** Desiree R. Delgadillo, Jessica L. Borelli, Emeran A. Mayer, Jennifer S. Labus, Marie P. Cross, Sarah D. Pressman

**Affiliations:** 1 UCLA G. Oppenheimer Center for Neurobiology of Stress & Resilience, CHS 42-210 10833 Le Conte Avenue, Los Angeles, CA 90095-7378 USA; 2 UCLA Vatche and Tamar Manoukian Division of Digestive Diseases, Los Angeles, USA; 3 David Geffen School of Medicine at UCLA, Health Sciences, Los Angeles, USA; 4 UCLA Goodman Luskin Microbiome Center, Los Angeles, USA; 5 University of California, Los Angeles, USA; 6 Department of Psychological Science, University of California, Irvine, USA; 7 Department of Psychology, University of Pittsburgh, Pittsburgh, USA

**Keywords:** Microbiology, Psychology, Biomarkers, Cardiology, Gastroenterology

## Abstract

Emerging research suggests that the gut microbiome plays a crucial role in stress. We assess stress-microbiome associations in two samples of healthy adults across three stress domains (perceived stress, stressful life events, and biological stress /Respiratory Sinus Arrhythmia; RSA). Study 1 (n = 62; mean-age = 37.3 years; 68% female) and Study 2 (n = 74; mean-age = 41.6 years; female only) measured RSA during laboratory stressors and used 16S rRNA pyrosequencing to classify gut microbial composition from fecal samples. Phylogenetic Investigation of Communities by Reconstruction of Unobserved States was used to predict functional pathways of metagenomes. Results showed differences in beta diversity between high and low stressful life events groups across both studies. Study 1 revealed differences in beta diversity between high and low RSA groups. In Study 1, the low perceived stress group was higher in alpha diversity than the high perceived stress group. Levels of *Clostridium* were negatively associated with RSA in Study 1 and levels *Escherichia/Shigella* were positively associated with perceived stress in Study 2. Associations between microbial functional pathways (L-lysine production and formaldehyde absorption) and RSA are discussed. Findings suggest that certain features of the gut microbiome are differentially associated with each stress domain.

## Introduction

The human gut microbiome is composed of bacteria, archaea, viruses, and fungi, and colonize virtually every surface of the planet, including the human gut. These non-human organisms have shaped human evolution from its origin with emerging research suggesting that they play crucial roles in not only physiological but psychological processes beginning as early as the prenatal period. Indeed, prenatal maternal stress is linked to microbial shifts^1–3^ associated with obesity and metabolic syndrome in adulthood^4^. Interestingly, research in laboratory animals has repeatedly revealed causal links between stress responses and microbial composition^5,6^. In humans, there are numerous studies showing associations between stress and the gut microbial composition, however, the number of studies showing causal links are limited^7,8^. Stress-microbiome research on healthy humans is scant and has largely focused on associations in clinical populations such as people diagnosed with stress-related disorders including depression and anxiety, or people diagnosed with brain-gut disorders such as irritable bowel syndrome^9^. Research that investigates stress-microbiome connections in healthy samples may highlight both therapeutic and detrimental components of the microbiome that could either support stress-resilience or, alternatively, increase stress vulnerability. Indeed, assessing stress-microbiome connections prior to the onset of pathology could contribute to the discovery of features in the gut microbiome that may prevent or delay the onset of stress-related disorders. Thus, the current work explores stress-microbiome connections in two samples of healthy adults to reveal possible patterns across samples and observe whether results replicate across these two studies.

Although stress is a multifaceted construct^10^, it is generally recognized as a condition in which environmental demands exceed one’s adaptive capacity, resulting in biopsychosocial changes that may increase disease risk^11^. Building upon this definition, stress can be described as being composed of three interrelated domains: objective environmental demand (e.g., major life events), psychological perceptions and appraisals of environmental demands, and biological responses to those demands (e.g., increases in blood pressure and cortisol) that result in physiological processes that can become nocuous to health^12^. While each of these domains represents an independent stress construct associated with health outcomes and indices^13–15^, these domains can also overlap to influence the stress experience. For example, the impact of stressful life events is influenced by psychological perceptions or appraisals (e.g., coping beliefs) of those experiences^16^. Similarly, without a robust physiological response to a stressful event or negative psychological perception, the downstream health impact may be negligible^15^. Indeed, these interlocking domains are correlated, but certainly not redundant^17^. Thus, it is valuable to explore each domain separately given that this may provide a better understanding of the stress-microbiome connection and underlying mechanisms. The current work explores relations between microbial composition and each stress domain. Namely, we assess associations between the gut microbiome, and indices of environmental stress (i.e., stressful life events), psychological stress (i.e., perceptions of stress), and heart rate variability/respiratory sinus arrythmia (RSA) in a reaction to a laboratory stressor (i.e., biological stress); hereafter referred to as stressful life events, perceived stress, and RSA stress reactivity.

Although environmental and psychological stress trigger an array of biological stress responses in the body, the current study focuses on the cardiovascular system, which can serve as a marker of autonomic nervous system stress reactivity. We are especially interested in heart rate variability (indexed by RSA), a marker of vagal tone and parasympathetic function, given its ties to stress^18–22^ as well as previously identified connections between vagal activity, gut function^23^ (e.g., motility, secretions, immune function), and microbial composition^24–26^.

The individual gut microbial profile is unique. There over two thousand different microbial species identified from the human gut which are classified into 12 different phyla^27^ (biological classification). In many ecosystems, including the human gut, a high diversity of species is a hallmark of a resilient, thriving environment, where many members of the system interact to promote stability^28,29^. In microbiome science, the diversity found within an ecosystem is called alpha diversity and the diversity between ecosystems is called beta diversity. In the human gut, higher alpha diversity is often associated with better health^30^. In contrast, lower alpha diversity is not only associated with several poor health outcomes such as diabetes, food allergies, asthma, and obesity, but it is also linked to deleterious psychological outcomes such as cognitive and mood disorders^28^, although the causal directionality of these associations in humans is not well-defined.

Together, the multitudes of diverse microorganisms in the gut form an ecosystem within the host that contain bacteria that can express symbiotic (non-harmful or beneficial to health) or pathogenic (detrimental to health) relationships with the host. Interestingly, variations of these pathogenic and symbiotic expressions are not only observed in physical health outcomes, but also psychological outcomes. For instance, higher emotion regulation was positively associated with levels of *Akkermansia* in children^31^ and the administration of *Akkermansia muciniphila* was shown to reduce depressive-like behaviors in mice exposed to chronic stress^31,32^. Additionally, some studies have found that those diagnosed with stress-related disorders have lower levels of *Lactobacillus* and *Bifidobacterium*^33,34^ when compared to healthy controls. Similarly, intervention studies have shown that the administration of certain strains of these bacteria alleviate psychological distress^35,36^. Other work has shown that higher levels of *Streptococcus*^37,38^, *Clostridium*^39,40^, and *Escherichia/Shigella*^41,42^ are positively associated with stress and stress-related disorders. See Table 1 for detailing of the previously referenced studies linking stress to *Lactobacillus, Akkermansia, Bifidobacterium, Streptococcus*, *Escherichia/Shigella*, and *Clostridium*. Further, it is important to note that some of the aforementioned genera contain both symbiotic and pathogenic species and strains. Thus, these complexities must also be considered when exploring stress-microbiome connections.

**Table 1:** Literature that informed the identification of select bacteria explored in association with stress domains.

Select bacteria	Article	Study description	Findings specific to select bacteria
*Lactobacillus*	Aizawa et al., 2016	Cross-sectional human study (N = 100; n = 43) comparing fecal microbiome samples of patients with major depressive disorder (MDD) to healthy controls	Patients with MDD had lower *Lactobacillus* counts than healthy controls
	Messaoudi et al., 2011	30-day clinical trial of healthy adults (N = 55; n = 26) who were administered probiotic formula *Lactobacillus helveticus* R0052 and *Bifidobacterium longum* R0175	Alleviated psychological distress in healthy adults compared to healthy controls
	Kim et al., 2020	*Escherichia coli* and *Lactobacillus mucosae* were isolated from healthy human feces and administered to mice (n = 7)	Oral gavage of *E.coli* caused depression in mice. Treatment with *L.mucosae* reduced *E.coli* induced depression
*Akkermansia*	Delgadillo et al., 2022	Cross-sectional study of human toddlers (N = 77) using fecal microbiome samples	*Akkermansia* was associated with increased emotion regulation in children
	Ding et al., 2021	Mice treated with *Akkermansia muciniphila* (AKK) for 3 weeks prior to chronic restraint stress (n = 6)	AKK treatment reduced depressive-like behaviors compared to untreated controls
*Bifidobacterium*	Aizawa et al., 2016	Cross-sectional human study (N = 100; n = 43) comparing fecal microbiome samples of patients with MDD to healthy controls	Patients with MDD had significantly lower *Bifidobacterium* counts compared to controls
	Liu et al., 2016	Cross-sectional human study (N = 100) comparing microbial composition (fecal samples) in IBS subgroups (n = 65), healthy controls (n = 20) and those with depression (n = 15)	Those with depression had significantly lower levels of *Bifidobacterium* compared to controls
*Streptococcus*	Gao et al., 2018	Mice were exposed to chronic stress for one month (n = 4–5)	Relative to normal mice, stressed mice showed increased levels of inflammation promoting bacteria, including *Streptococcus*
	Nishida et al., 2019	Japanese medical students (N = 60; n = 31) were randomly assigned to a placebo or probiotic treatment (1 tablet taken daily for 24 weeks) group prior to the national examination for medical practitioners. Fecal samples were collected pre and post treatment	Levels of *Streptococcus* were elevated in the placebo group compared to the control group
*Escherichia/Shigella*	Bashir et al., 2024	Cross-sectional study of healthy adult women (N = 160) measuring symptoms of stress, depression, fecal, and vaginal microbiome	The relative abundance of *Escherichia* and *Shigella* were elevated in women with greater depression symptoms
	Chen et al., 2021	Cross-sectional study of adult female patients (N = 108; n = 62) comparing fecal microbiome samples of patients with MDD to healthy controls	Patients with MDD had higher levels of *Escherichia/Shigella* than healthy controls
*Clostridium*	Bailey et al., 2011	Mice (n = 5) were exposed to a 2-hour social defeat stressor for six days and cecal microbiota were assayed	Stressor exposure increased relative abundances of *Clostridium*
	Mullie et al., 2002	Fecal microbiome samples were collected from healthy males (N = 10) six weeks prior to and during final examinations	A rise in *Clostridium perfringens* was observed during the examination period

*Note.* Both total sample size (N) and treatment group sample size (n) are reported in human studies when applicable.

Interestingly, microbes found in the human gut perform various functions in hosts, including the production of stress reducing metabolites. For instance, *Intestinimonas* strain AF211 can convert the essential amino acid L-lysine into the short chain fatty acid butyrate^43^. Butyrate is a metabolite that can cross the blood brain barrier, however, given that most butyrate is metabolized by colonocytes and the liver, it may be more likely that butyrate indirectly influences the brain by regulating vagus nerve activity^44^. Supplementation of this metabolite reduces inflammation, provides neuroprotection, improves behavioral symptoms^45^, and attenuates the cortisol response to psychosocial stress^46^. Indeed, a review of 24 human studies showed robust evidence that dietary supplementation of L-lysine and L-arginine (amino acid) effectively treated anxiety-related disorders such as depression and generalized anxiety^47^. Specifically, L-lysine and L-arginine supplementation reduced trait anxiety, stress-induced anxiety, and basal levels of salivary cortisol in a double-blind placebo-controlled study of 108 healthy human adults^48^. In contrast, gut microbiota can also excrete toxins that can cross the blood–brain barrier such as formaldehyde. Formaldehyde has been shown to contribute to age-related cognitive impairment and neurodegenerative diseases such as Alzheimer’s Disease^49–51^. Diseases such as these can trigger the onset of stress-related conditions such as depression, anxiety, and aggression^49^.

The current work is the first to assess whether microbial alpha and beta diversity, select microbes (see Table 1), and microbial function (e.g., produce or aid in the absorption of amino acids or metabolites) might be linked differentially to stress. Specifically, we explore whether three indices of stress (perceived stress, stressful life events, and RSA in response to acute stress) differentially relate to alpha and beta diversity, select microbes, and microbial function in the gut microbiome. It is important to note that the research referenced in Table 1 linking stress to the select bacteria includes animal research, intervention studies, and cross-sectional human research. In the current study, we use these studies to explore whether similar stress-microbe links can be observed in the endogenous gut microbiome in healthy human samples. These findings may provide insight when designing specific stress intervention studies that could promote an increase in abundances of microbes linked to mitigated stress. Further, given that there is no published work examining environmental, psychological, and biological stressors across multiple samples of healthy humans, these findings could add crucial knowledge to the growing stress-microbiome literature. Please see the methods section for a detailed account of procedures in Study 1 and Study 2. In brief, we use self-reports to measure stressful life events^52^ and perceived stress^53^. RSA is measured using ECG equipment during a laboratory stressor and stool samples are collected by participants at one time-point. These overarching methods were common in both studies.

## Results: Study 1

### Descriptive statistics

The subset that completed the microbiome portion of the study consisted of 62 adults between the ages of 25–65. The sample was 68% female with a mean age of 37.3 years. When asked about their race/ethnicity, 32.3% identified as White, 29% identified as Asian, 21% declined to answer, 9.7% identified as Hispanic, 3.2% identified as Black, and 4.8% identified their race/ethnicity as “other.” Please see Table 2 for a summary of stress group membership by sex.

**Table 2:** Summary of stress group membership by sex in Study 1.

Stress group	Males	Females	Total
Low perceived stress	8	14	22
Mid perceived stress	6	15	21
High perceived stress	3	16	19
Low stressful life events	6	14	20
Mid stressful life events	5	17	22
High stressful life events	6	14	20
Low reactivity RSA	4	16	20
Mid reactivity RSA	4	17	21
High reactivity RSA	9	12	21

### Stressful life events

#### Stressful life events and beta diversity in Study 1

**Fig. 1:**
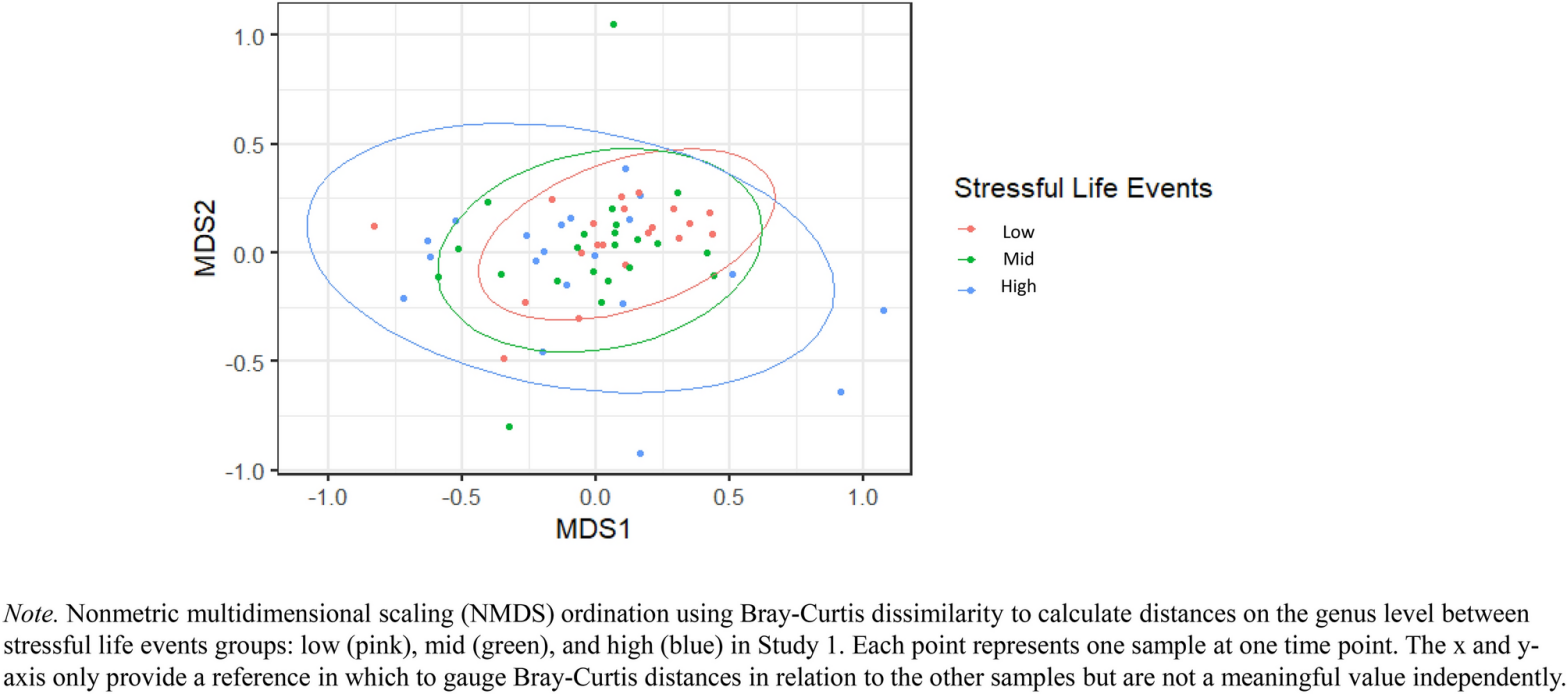
Differences in Beta Diversity between Low, Mid, and High Stressful Life Events Groups in Study 1.

A PERMANOVA revealed that there were significant differences in community structure between high, mid, and low stressful life events groups in Study 1, *F*(2,59) = 1.70, *R*^2^ = 0.06, *p* = 0.03 (Fig. 1).

After controlling for sex, *F*(1,58) = 1.93, *R*^2^ = 0.03, *p* = 0.04, the results remained significant, *F*(1,58) = 1.73, *R*^2^ = 0.06, *p* = 0.02. Specifically, there were group differences between the low and high stressful life events groups, *F*(1,38) = 2.12, *R*^2^ = 0.05, *p* = 0.03, but not the low and mid groups, *F*(1,40) = 1.17, *R*^2^ = 0.03, *p* = 0.28, or mid and high groups, *F*(1,40) = 1.76, *R*^2^ = 0.04, *p* = 0.06 (see Supplemental Table 1).

#### Differential abundances of microbes in stressful life events groups

In Study 1, *Intestinimonas* and *Faecalibacterium/Subdoligranulum* were absent/undetectable in the high stressful life events group. Additionally, *Eubacterium/Roseburia* was absent/undetectable in the high stressful life events groups. A complete report of present/absent outcomes for stressful life events are found in Supplemental Table 2.

There were no statistically significant differences in alpha diversity between low, mid, and high stressful life events groups (see Supplemental Table 3). Further, there were no statistically significant associations between stressful life events and select bacteria (see Supplemental Table 4).

### Perceived stress

#### Perceived stress and alpha diversity in Study 1

**Fig. 2:**
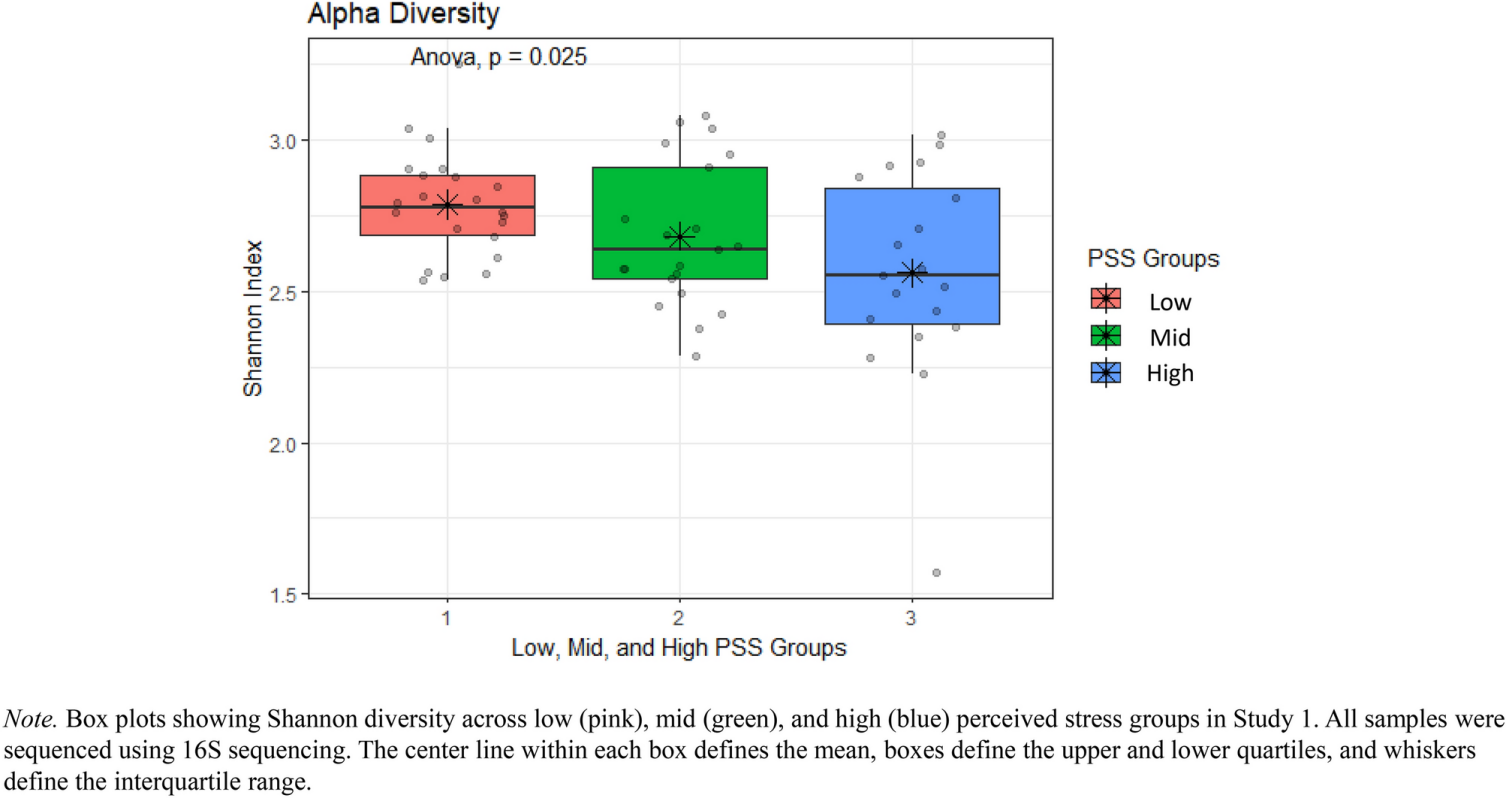
Shannon Diversity of those in the Low Perceived Stress Group is Significantly Higher than those in the High Perceived Stress Group.

A one-way ANOVA revealed statistically significant group differences in alpha diversity between perceived stress groups (low, mid, and high) in Study 1, *F*(2, 59) = 3.92, *p* = 0.03 (Fig. 2).

A one-way ANCOVA determined that this difference remained after controlling for age, sex, BMI, diet, and general health, *F*(2, 52) = 5.04, *p* = 0.01. Specifically, Tukey’s HSD Test for multiple comparisons found that the mean value of alpha diversity was significantly different between low and high stress groups (adj.* p* = 0.03, 95% C.I. = -0.42, -0.02). There were no statistically significant differences between low and mid stress groups (adj*. p* = 0.50) or mid and high stress groups (adj.* p* = 0.31).

There were no statistically significant differences in beta diversity between low, mid, and high perceived stress groups (see Supplemental Table 1). Additionally, there were no statistically significant associations between perceived stress and select bacteria (see Supplemental Table 4).

### RSA stress reactivity

#### RSA stress reactivity and beta diversity in Study 1

**Fig. 3:**
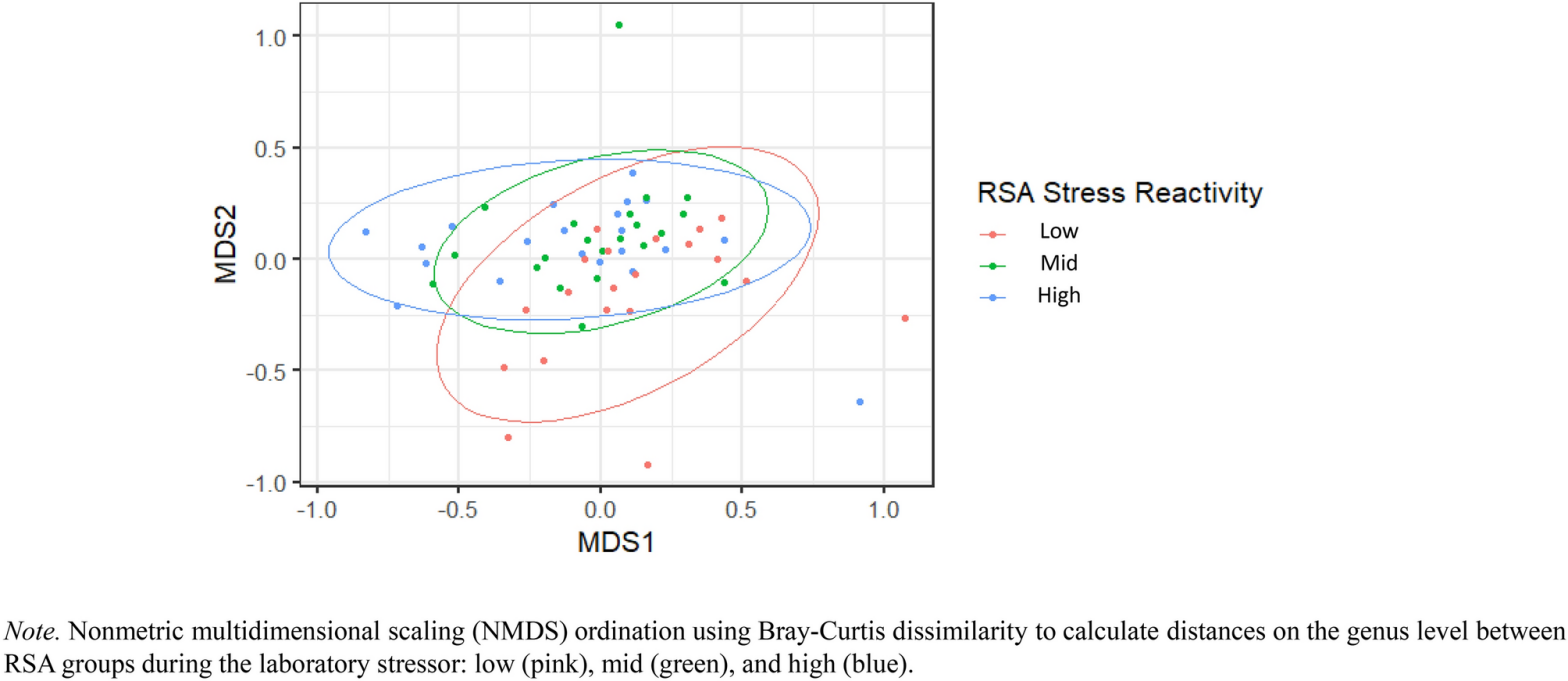
Differences in Beta Diversity between Low, Mid, and High RSA Stress Reactivity Groups in Study 1.

PERMANOVA revealed differences in beta diversity between RSA stress reactivity groups in Study 1 in response to the laboratory stressor. There were significant differences in community structure between high, mid, and low RSA reactivity groups, *F*(2,59) = 2.13, *R*^2^ = 0.07, *p* = 0.01 (Fig. 3).

After controlling for sex (*R*^2^ = 0.02, *p* = 0.13) and baseline RSA (*R*^2^ = 0.01, *p* = 0.81), results remained significant, *F*(2,57) = 2.14, *R*^2^ = 0.07, *p* = 0.01. Further, PERMANOVA revealed differences between the high and low RSA groups, *F*(1,39) = 2.65, *R*^2^ = 0.07, *p* = 0.004, and the low and mid RSA groups, *F*(1,39) = 2.67, *R*^2^ = 0.06, *p* = 0.01, but not the mid and high RSA groups, *F*(1,41) = 1.04, *R*^2^ = 0.03, *p* = 0.37.

#### Differential abundances of microbes in RSA stress reactivity groups

The stress reactivity groups differed with respect to presence/absence of bacteria (see Supplemental Table 5) as well as abundance of certain taxa. Specifically, the low RSA reactivity group had higher levels of *Eggerthella* than the mid RSA reactivity group (lfc = -5.23; adj. *p*-value = 0.04) and the high RSA reactivity group (lfc = -5.25; adj. *p*-value = 0.02). Further, the low RSA reactivity group was significantly lower in *Lachnoclostridium/Roseburia* (lfc = 5.91; adj. *p*-value = 0.02) than the high RSA reactivity group.

#### Associations between levels of RSA stress reactivity and select microbes

Pearson correlations were performed to reveal associations between select bacteria and levels of RSA in reactivity to the acute laboratory stressor. *Clostridium* was significantly negatively associated with RSA stress reactivity, *r*(62) = -0.37, *p* = 0.003 (see Supplemental Table 6). Hierarchical regression was then performed adjusting for sex, diet, overall health, BMI, age, and baseline RSA (adjusted *R*^*2*^ = 0.09, *p* = 0.10) and *Clostridium* remained negatively associated with RSA stress reactivity, Δ*R*^*2*^ = 0.08, *b* = -0.48, *SE* = 0.21, *p* = 0.02, 95% CI = -0.89 to -0.07. As a reference, the mean relative abundance value of *Clostridium* (prior to CLR transformation) was 0.49% and the standard deviation was 1.35%.

#### RSA stress reactivity and differential microbial functional pathways

The high RSA stress reactivity group was significantly higher in pathway abundances related to the production (biosynthesis) of L-lysine compared to the low group, *B* = -1.04, SE = 0.29, *p* < 0.001, *q* = 0.21, *d* = -1.59, 95% CI =-2.51 to -0.66, after controlling for physical covariates. Additionally, association analysis revealed that higher levels of pathway abundances related to formaldehyde (absorption) assimilation were associated with lower levels of RSA stress reactivity, *B* = –0.57, SE = 0.21, *p* < 0.001, *q* = 0.14, after controlling for physical covariates.

There were no significant differences in alpha diversity between low, mid, and high RSA groups (see Supplemental Table 3). Further, except for *Clostridium* reported previously, there were no statistically significant associations between RSA and select bacteria (see Supplemental Table 4).

## Results: Study 2

### Descriptive statistics

Seventy-four healthy female participants completed the current study (*M*_age_ = 41.6 years, SD_age_ = 6.2 years; 55.6% identified as White, 23.6% identified as Asian, 11.1% chose more than one race/ethnicity, 7% identified their race/ethnicity as “other,” and 2.8% identified as Native Hawaiian). Please see Table 3 for a summary of stress group membership.

**Table 3:** Summary of stress group membership in Study 2.

Stress group	Total (females only)
Low perceived stress	26
Mid perceived stress	23
High perceived stress	25
Low stressful life events	24
Mid stressful life events	25
High stressful life events	25
Low reactivity RSA	24
Mid reactivity RSA	25
High reactivity RSA	25

### Stressful life events

#### Stressful life events and beta diversity in Study 2

**Fig. 4:**
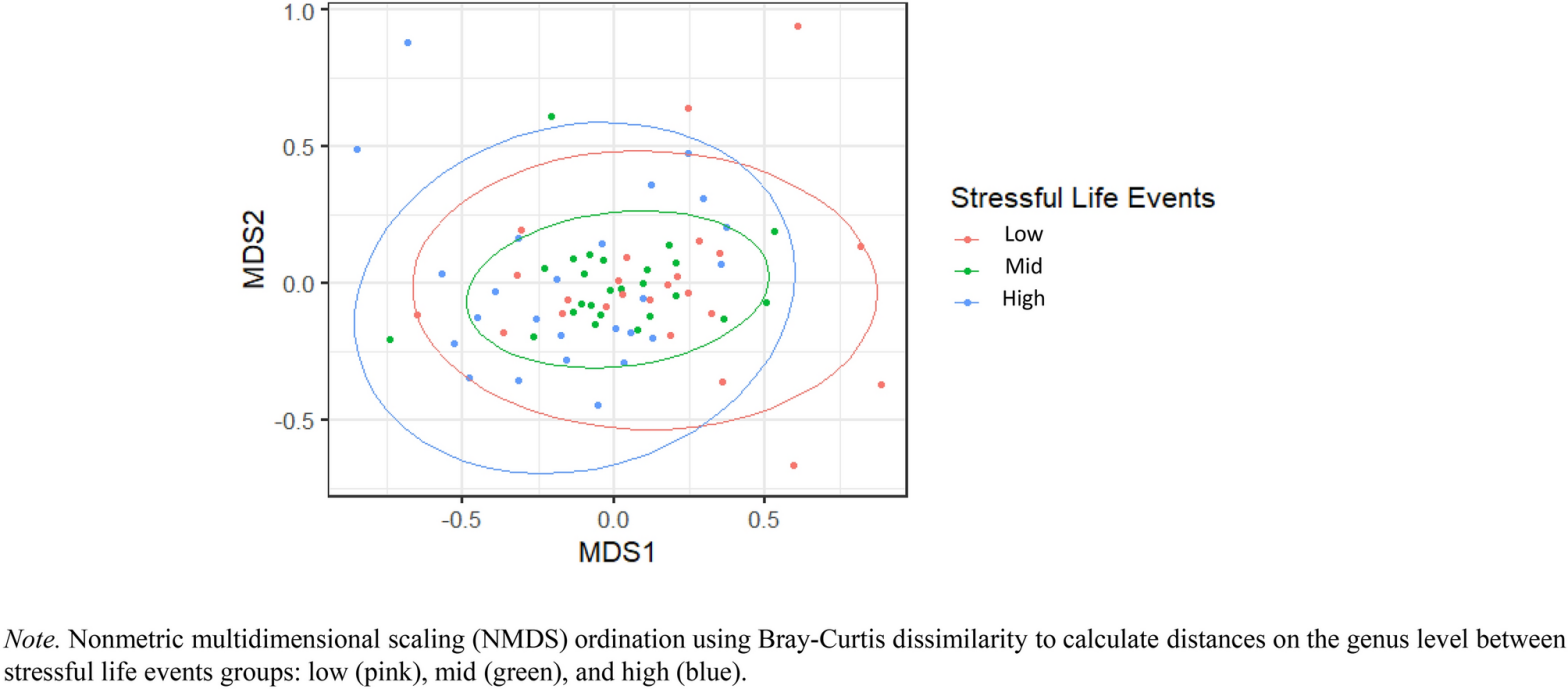
Differences in Beta Diversity between Low, Mid, and High Stressful Life Events Groups in Study 2.

Study 2 also showed differences in beta diversity in stressful life events groups. PERMANOVA revealed that there were significant differences in community structure between high, mid, and low stressful life events groups, *F*(2,71) = 1.88, *R*^2^ = 0.05, *p* = 0.01 (Fig. 4).

Specifically, Study 2 results revealed group differences between the low and high stressful life events groups, *F*(1,47) = 2.05, *R*^2^ = 0.04, *p* = 0.03, and between the mid and high groups, *F*(1,48) = 2.21, *R*^2^ = 0.04, *p* = 0.02, but there were no statistically significant differences between the low and mid groups, *F*(1,47) = 1.34, *R*^2^ = 0.03, *p* = 0.21.

#### Differential abundances of microbes in stressful life events groups

Low, mid, and high stressful life events groups showed differences in the presence and abundance of certain microbes and significantly differed in absolute abundances of certain genera between stressful life events groups. *Marvinbryantia* was higher in the low stressful life group compared to the high group (lfc = -3.760; adj *p* = 0.039). *Flavonifractor* was higher in the high stressful life events groups compared to the low stressful life events group (lfc = 5.124; adj. *p* = 0.039, Additionally, *Eubacterium, Lactobacillus, and Shuttleworthia* were absent/undetectable in the high stressful life events group. For a complete list of bacteria absent/undetectable in stressful life events groups, see Supplemental Table 7.

There were no statistically significant differences in alpha diversity (see Supplemental Table 8) or beta diversity (see Supplemental Table 9) between low, mid, and high perceived stress groups. Further, there were no statistically significant associations between stressful life events and select bacteria (see Supplemental Table 10).

### Perceived stress

#### Perceived stress and *Escherichia*/*Shigella* in Study 2

Levels of *Escherichia/Shigella* were significantly and positively associated with perceived stress in Study 2, *r*(72) = 0.26, *p* = 0.02 (see Supplemental Table 11). After adjusting for covariates (adjusted *R*^*2*^ = 0.10, *p* = 0.03), *Escherichia/Shigella* remained significantly, positively associated with perceived stress, Δ*R*^*2*^ = 0.05, *b* = 0.16, *SE* = 0.88, *p* = 0.049*,* 95% CI = 0.01—3.53. The mean relative abundance of *Escherichia/Shigella* (prior to CLR transformation) was 0.15% with a standard deviation of 0.37%.

There were no statistically significant differences between perceived stress groups for alpha diversity (see Supplemental Table 8) or beta diversity (see Supplemental Table 9). Further, except for *Escherichia/Shigella,* there were no significant associations between perceived stress and select bacteria (see Supplemental Table 10).

## RSA stress reactivity

### RSA stress reactivity and *Lactobacillus*

*Lactobacillus* was significantly positively associated with RSA stress reactivity (r(72) = 0.269, *p* = 0.020). After adjusting for age, BMI, diet, health, and baseline RSA (adjusted *R*^*2*^ = 0.642, *p* < 0.001), *Lactobacillus* was no longer significantly associated with RSA stress reactivity, Δ*R*^*2*^ = 0.013, *b* = 1.768, *SE* = 0.098, *p* = 0.110, 95% CI = -0.037—0.353**.**

There were no statistically significant differences in alpha diversity (see Supplemental Table 8), or beta diversity (see Supplemental Table 9) between low, mid, and high RSA stress reactivity groups. Additionally, there were no statistically significant associations between levels of RSA and select bacteria (see Supplemental Table 10) (Table 4).

**Table 4:** Associations and group differences in microbial composition by stressor type for Study 1 and Study 2.

Stress measures	Study 1	Study 2
**Alpha diversity**		
Perceived stress	Increased in low compared to high	x
Stressful life events	x	x
RSA stress reactivity	x	x
**Beta diversity**		
Perceived stress	x	x
Stressful life events	Group differences	Group differences
RSA stress reactivity	Group differences	x
**ANCOM-BC**		
Perceived stress	x	x
Stressful life events	Differential abundances between groups (present/absent)	Differential abundances between groups (present/absent) *Marvinbryantia *↓ in high group *Flavonifractor* ↑ in high group
RSA stress reactivity	Differential abundances between groups (present/absent) *Eggerthella * ↓ in high RSA group *Lachnoclostridium/Roseburia * ↓ in low RSA	x
**Regressions**		
Perceived stress	x	*Escherichia/Shigella* positively associated with perceived stress
Stressful life events	x	x
RSA stress reactivity	*Clostridium* negatively associated with RSA	x

*Note.* This table represents broad findings and displays a comparison of results across Study 1 and Study 2 with ‘x’ depicting null findings. Details of differential abundances (present/absent) results can be found in Supplemental Table 2, 5, and 7.

## Discussion

Associations between stress and microbiota appeared in three different stress domains. While most previous studies have examined the impact of individual stressors or stress-related conditions such as depression and anxiety on the microbiome, our work takes a more comprehensive stress-focused approach in two samples of healthy adults. The nature of these connections varied across domains. For instance, there were significant differences in microbial composition (beta diversity) between stressful life events groups. Specifically, stressful life events explained approximately 5% of microbiota variance between high and low stress groups in both studies revealing a relatively robust, replicable finding. To provide context, diet explains about 5 to 20% of gut microbiota variation^54^, while host genetics explains about 1.9 to 8.1% of gut microbiome variation^55,56^. These replicated findings suggest that stressful life events may have comparable importance on the composition of the gut microbiome as diet or genetics. Next, we showed associations between composition of the gut microbiome and perceived stress. In Study 1, individuals who were low in perceived stress had higher alpha diversity compared to those with high perceived stress. In Study 2, levels of the select pathogen *Escherichia/Shigella* were positively associated with perceived stress. Within the context of biological stress, Study 1 revealed differences in beta diversity between high and low RSA stress reactivity groups and showed that higher levels of the select pathogenic bacteria *Clostridium* predicted lower levels of RSA stress reactivity. Finally, we found that the high RSA group was significantly higher in functional pathways related to L-lysine biosynthesis compared to the low RSA group and that functional pathways related to formaldehyde assimilation were negatively associated with levels of RSA during the acute laboratory stressor.

Significant differences in microbial composition (beta diversity) between stressful life events groups were demonstrated in *both* studies, suggesting that stressful life events play a governing role in shifting microbial composition. Stressful life events may reshape or perhaps redistribute populations of bacteria that live in the gut. Via their lasting and robust effects on everyday life, stressful life events may trigger a repeated influx of stress hormones to which the gut microbiome has not yet become habituated. Indeed, microbial growth and colonization is altered by mammalian stress hormones such as epinephrine and norepinephrine^57^ and, thus, may be more likely to influence microbial composition across sample types.

Exploratory analyses revealed that the abundances of numerous microbes were absent/undetectable in the high stressful life events groups in both studies; however, exhaustive inferences detailing the role that each microbe might play in the brain-gut-microbiome system is beyond the scope of extant literature, as many of the microbes listed in Supplemental Tables 2, 5, and 7 have not yet been evaluated within the context of stressful life events. Nevertheless, a few potentially important stress-associated microbes will be discussed. For instance, *Eubacterium/Roseburia* were absent/undetectable in the high stressful life events group in Study 1. Notably, some research suggests that these are butyrate producing, health supporting bacteria. Indeed, certain species of *Eubacterium* are thought to contribute to gut health by reducing inflammation in the gut^58^. Similarly, *Roseburia* is also thought to have therapeutic, inflammation reducing properties in the gut. Both *Eubacterium* and *Roseburia* include several butyrate producing species. Butyrate is a metabolite that plays a crucial role in the suppression of inflammation^58^, which supports gut barrier integrity^59^. Importantly, evidence suggests bidirectional links between *Eubacterium/Roseburia* and stress^60,61^. For example, Xu and colleagues (2021) found that, following chronic stress, gut epithelial integrity was restored in rats treated with *Roseburia*^61^, and another study demonstrated that rats in chronic stress showed decreases in levels of *Roseburia*^60^.

There were significant differences in alpha diversity between high and low perceived stress groups for Study 1, such that those lower in perceived stress had greater alpha diversity (i.e., a more diverse microbiome) than those in the high perceived stress group. These results support other studies connecting lower levels of perceived stress to increased alpha diversity^62–64^. One bottom-up explanation for this is that microbial alpha diversity in the gut may not only support better health outcomes but may also play a role in how we appraise stress. Specifically, prebiotics (e.g., fiber) have been shown to stimulate the growth of endogenous commensal gut bacteria^65^, such as *Bifidobacterium* and *Lactobacillus,* and to reduce negative emotions. Neuroactive metabolites produced by these microbes could reduce stress responsiveness^66^. A top-down explanation for the findings between perceived stress and alpha diversity is that psychological perceptions of threat can lead to measurable changes in physiological processes in the gastrointestinal system^67^. Specifically, heightened appraisals of threat lead to stress-induced alterations in gut motility, secretions, and permeability^67,68^. Alterations may lead to shifts in gut microbial composition, in the microbiomes of both patient populations and healthy adults^69^.

*Escherichia/Shigella* were significantly positively associated with perceived stress in Study 2 and these results persisted even after controlling for physical covariates. These findings complement other work linking higher levels of *Escherichia/Shigella* to post-traumatic stress disorder^70^ and anxiety^71^. Indeed, pathogenic bacteria, including certain strains of *Escherichia/Shigella*, are linked to negative health outcomes such as gut dysbiosis and irritable bowel syndrome^72^. These bacteria are also linked to more severe stress responses across multiple stressor types, including psychological and biological stress^73–77^. Further, perceived stress is associated with biomarkers of inflammation^78–80^. Thus, one reason for the positive associations between perceived stress and *Escherichia/Shigella* could be that those high in *Escherichia/Shigella* did not report or were unaware that they were ill or on the verge of illness at the time of the study and, thus, were not screened out of participation. It is possible that underlying illness led them to report more negative affect related constructs, including a higher perception of stress.

Finally, there were differences in beta diversity across different RSA stress reactivity groups in Study 1. As described previously, high RSA (at rest) is linked to better psychological wellbeing while low RSA is linked to poorer psychological wellbeing^22^. Interestingly, analyses revealed that *Slackia* was only absent/undetectable in the low RSA stress reactivity group. *Slackia* is a potentially protective genus that produces the metabolite dihydroresveratrol (DHR), which was shown to have anti-cancer and anti-inflammatory effects in mice^81^. Further, *Slackia* is negatively associated with cortisol and psychological distress (daily hassles) in humans^3^. Similarly, *Blautia* was only absent/undetectable in the low RSA reactivity group and is another butyrate producing bacteria. *Blautia* is negatively correlated with visceral fat and is thought to mitigate metabolic syndrome^82^, but no work, to our knowledge, has connected it with RSA stress reactivity. Interestingly, a recent mindfulness-based intervention was found to significantly increase levels of *Blautia* in pregnant women^83^, suggesting that there may be connections between stress reduction and this bacterium, and that this direction may be a promising avenue for future research.

Next, higher levels of *Clostridium* were associated with lower levels of RSA reactivity, even after controlling for physical covariates. In relation to stress, one strain of *Clostridium* was significantly increased in students during an examination period^40^. However, other strains of *Clostridium* are considered commensal, produce butyrate, and are thought to have probiotic properties^84^. This emphasizes the fact that gut microbes exist within an ecosystem and symbiotic versus pathogenic roles of gut bacteria likely depend on checks and balances such as genetics and populations of other bacteria that exist within the context of each specific ecosystem. Since the current study found that *Clostridium* predicts lower RSA stress reactivity, this could mean that the particular strains of *Clostridium* linking the two variables may play a pathogenic role within the context of cardiovascular (RSA) function during acute stress.

Interestingly, we discovered that the high RSA stress reactivity group was significantly higher in metagenomic functional pathway abundances related to the biosynthesis of L-lysine, an amino acid that can be converted to butyrate. Established research repeatedly shows that L-lysine supplementation is linked to mitigated stress-related conditions and processes such as trait anxiety, stress-induced anxiety, and basal levels of salivary cortisol^47,48^. The current work may be the first to demonstrate that those with high levels of RSA during acute stress have higher levels of L-lysine compared to those with low levels of RSA during acute stress. Since high RSA is a proxy for PNS activity, it is possible that L-lysine helps to dampen SNS activity and support PNS activity via the vagus nerve. We also discovered that higher levels of pathway abundances related to formaldehyde assimilation were associated with lower levels of RSA during stress reactivity. Since high levels of formaldehyde are linked to age-related cognitive impairment^49–51^, it is possible that the deleterious effects of this toxin extend beyond neurological function into cardiovascular function, particularly in response to acute stress. Collectively, these findings suggest that some microbial metabolites could mitigate cardiovascular stress responses while others exacerbate these responses during acute stress.

Putting the above findings together, the differences in both microbial composition and function in RSA reactivity groups and levels of RSA complement established research that reveals connections between higher RSA and better social and emotional function^85^ and the ability to respond to environmental demands^86,87^. Specifically, the current work showed that those with lower levels of RSA stress reactivity had higher levels of *Clostridium* and greater abundances of pathways linked to formaldehyde absorption. We further demonstrate that those with higher RSA during acute stress differed from those with lower RSA during acute stress in microbial function (i.e., higher L-lysine biosynthesis in the high RSA group). As discussed previously, the afferent vagus nerve can transmit signals that can exacerbate or mitigate anxiety depending on the bacterial stimulus^24^. Thus, the vagus nerve may be a key mediator in these findings and should be considered in future research assessing various types of stress.

This study was cross-sectional, limiting causal inferences. Additionally, samples were not collected simultaneously (e.g., some fecal samples were collected weeks after laboratory stress visits), allowing for potential third factors such as other stressors to influence results^88^. Relatedly, Study 1 and Study 2 took place during different periods of time, with Study 1 overlapping with the COVID-19 pandemic, and Study 2 not overlapping. Since COVID-19 related quarantine protocols have been linked to altered microbial composition^89^, this may have impacted the replication of findings between studies, as could the differential characteristics of the samples (i.e., parents vs. non-parents, mixed sex sample vs. women only). Specifically, adequately powered experimental designs and prospectively recruiting larger samples of men and women based on rigorous power analyses are an important direction for future research. Further, the sample size limited the number of confounders included in statistical models, thus, some variables such as SES were not included as covariates. Finally, 16S amplicon sequencing cannot identify or quantify individual microbial strains. This is an important limitation because there is a strong possibility that links between stress and the gut microbiome are more closely tied on the strain level as opposed to the genus level. Moving forward, researchers should consider using shotgun metagenomic sequencing which accurately identifies microbes on the strain level and is, thus, regarded as the gold standard measure. Additionally, future microbiome research assessing stress domains should be conducted experimentally and longitudinally (e.g., by measuring microbial alterations pre and post experimental stress exposure or via prospective longitudinal studies with the microbiome assessed repeatedly over time in conjunction with stress experiences) in samples that are more similar and occur during the same year and should include shotgun metagenomic sequencing.

The current work is the first to explore connections between stressful life events, perceived stress, and RSA stress reactivity surrounding acute stress, and microbial composition in two samples of healthy adults. Each stress domain reflects distinct but overlapping components of stress, with perceived stress reflecting everyday stress appraisals and psychological constructs related to negative affect, RSA stress reactivity reflecting an acute biological response to a moderate social stressor, and stressful life events reflecting more objective environmental stressors that can be serious with lasting consequences (e.g., death of a spouse, loss of a job).

Findings suggest that the gut microbiome could hold both therapeutic and nocuous properties that impact psychological and physiological stress and lend support to the concept that the gut microbiome, like other body systems, may also bear an allostatic load. The current studies lay the groundwork for the discovery of a counterpart to a stress-bearing allostatic load that we describe as a *therapeutic reservoir* housed in the gut. Future research should focus on designing experimental studies and longitudinal interventions that can identify both top-down and bottom-up pathways that promote mutual wellbeing in the brain-gut-microbiome system. Further, interventions that mobilize these beneficial bidirectional connections could promote wellbeing in both healthy and patient populations across races/ethnicities and socioeconomic statuses.

## Method: Study 1

### Participants

The current study assessed a subset of participants from a larger study on stress, emotion, and health indices. Participants in the larger study were recruited through Craigslist, Facebook, retirement homes, flyers, email, and class announcements (for undergraduate and graduate students at a large Western university) in the United States. Individuals who participated in the laboratory portion of the larger study were contacted via email from May 2020 through October 2020. The recruitment email contained a $5 gift code as a thank you gift for previous participation and 143 individuals were invited to take part in the at-home microbiome portion of the study and 62 were included in analyses (see Supplemental Fig. 1 for details). Participants were compensated $150 for the original larger study and an additional $25 for participation in the microbiome portion follow-up. Only four participants reported taking antibiotics within the past month and the remaining participants reported taking them within the past 6 months or longer (i.e., within the last year, 5 years or longer) and none of these were excluded. Individuals were excluded if they were pregnant; had a chronic pain condition; had pulmonary disease, neurological, or psychiatric disorder; had a clinical disorder such as depression or anxiety; had a history of cardiovascular disorder (including hypertension); smoked at the time of recruitment; or regularly took mood altering prescription medication, pain altering medication (e.g., Tylenol, Aspirin), cardiovascular function altering medication (e.g., antidepressants, beta blockers, blood pressure altering medication, amphetamines), or four or more medications. Non-English speakers, those who could not perform study tasks, and those who did not own a smartphone were also excluded. The study was approved by the Institutional Review Board (IRB) at the University of California, Irvine (HS# 2017–3516). We confirm that all experiments were performed in accordance with IRB guidelines and regulations.

### Procedures

There were three distinct parts of the current study: baseline assessment, stress reactivity session, and home microbiome assessment. Participants were screened online for study eligibility prior to being scheduled for participation and an informed consent was obtained from all the participants during their baseline assessment. Approximately one week following the baseline assessment, participants completed the in-lab stress reactivity session. Following a series of questionnaires, participants were connected to an electrocardiogram (ECG). Resting baseline RSA were collected for 6 min while the participant sat quietly. During this time, participants were instructed to sit still, sit up straight, keep their legs and arms uncrossed, and breathe normally.

Participants then completed the Trier Social Stress Test^90^ (TSST), a standardized stressor task, in front of a video camera and one judge (research assistant). For this task, participants were given two minutes to prepare a speech in which they would create an argument explaining why they would be the best candidate for a leadership position at their place of work, club, or organization that they were a part of, and then delivered the speech over three minutes in front of the “judge” (a confederate research assistant) and while on camera. Participants were told that their performance would be analyzed by the judge, who was an expert trained in public speaking. If the speech was under three min, the judge informed the participant that the three minutes were not completed and reminded them that they must fill up the entire three minutes. Participants were permitted to pause, but if they did not continue after 20 s, they were told by the judge that they must continue speaking. During the speech, the judge would say planned critical phrases like, *“You are being too superficial. Please provide additional examples”* and *“You are spending too much time on this aspect; please move on to another strength.”* RSA measures were taken throughout the tasks.

Immediately after the speech, the two-minute math task began. The participant was instructed to subtract the number 13 from 1,022 and report their answer verbally. They were told to start over if any mistakes were made. Their time began immediately after the instructions were given and if the participant reported an incorrect answer or was speaking too slowly, the researcher was instructed to say, *“That is incorrect, please start over from 1,022”* and “*Please go as fast as possible.”*

Participants who completed the baseline assessment and the stress reactivity session were invited by email weeks to months later to participate in the home microbiome portion of the study. The recruitment email contained a $5 Amazon credit code and a thank you for prior involvement. There was no obligation to join the home microbiome session to receive the initial $5; however, participants were informed that they would be compensated with another $20 for their continued involvement. Those who agreed were sent an online study information sheet and questionnaires measuring self-reported stress, diet, and health. They were also mailed a fecal collection kit with detailed instructions designed for public use. All participants were asked not to make any major dietary changes prior to the collection of the microbial sample and to collect and mail the sample within 1–2 weeks of receiving the collection kit. Participants were provided with a pre-paid, pre-addressed box to return the biological specimen according to official United States Postal Service standards. Following at-home collection, the fecal sample was mailed to the researchers for storage and was later sent to another institution and assayed for microbial composition.

## Measures

### Perceived Stress Scale (PSS)

The current study measured perceptions of psychological stress using the Perceived Stress Scale^91^ (PSS-10). This 10-item measure assessed the frequency in which individuals perceived stress in the last month on a scale from 0–4. The scale includes items such as, “In the last month, how often have you been upset because of something that happened unexpectedly?” and, “In the last month, how often have you been able to control irritations in your life?”, for which participants select “never,” “almost never,” “sometimes,” “fairly often,” or “very often.”

### The Holmes-Rahe Life Stress Inventory

The Holmes-Rahe Life Stress Inventory^52^ was used to measure exposure to environmental stress. This survey rated 43 potentially stressful life events that individuals may have experienced in the past year. Participants were asked to read through a checklist and answer “yes” or “no” regarding whether or not they had experienced occurrences such as “the death of a spouse,” “retirement,” or a “change in financial state.”

### Demographics and Body Composition Questionnaires

Participants self-reported demographics (race/ethnicity, education, and income). Weight and height data were collected by a trained nurse. Participant’s height and weight were used to calculate body mass index (BMI).

### Short Form Health Survey (SF-36)

The Short Form Health Survey^92^ (SF-36) is a commonly administered 36-item quality of life measure. Participants were asked to report on their health status by rating their health on a scale from 1–5, as “Excellent” to “Poor.” Specific questions pertaining to general health ratings, bodily pain, and physical and mental health issues limiting one’s occupational and social activities were asked. Items were rated on a Likert scale. For example, the scale asks the participants, “Compared to one year ago, how would you rate your health in general now?” with answers ranging from 1–3, “much better now than one year ago” to “much worse now than one year ago.''.

### Rapid Eating Assessment for Participants (REAP-S)

The Rapid Eating Assessment for Participants scale^93^ is a survey that assesses nutrition and physical activity and dietary patterns. and was used to measure fruit/vegetable, meat, and grain consumption as dietary covariates in the current study. In the current study, diet was defined as the number of servings consumed per week of fruit/vegetable, meat, and grain consumption. Questions include, “In an average week, how often do you eat less than 2 servings of whole grain products or high fiber starches a day?” and, “In an average week, how often do you eat less than 2 servings of vegetables a day?” Answer choices were “usually/often,”* “*sometimes,”* “*never,” and “does not apply to me.”

### Cardiovascular

Biological stress was assessed using measures of RSA at rest and in reactivity to the laboratory stressor. RSA was collected using ECG and impedance cardiogram (ICG) equipment. Five disposable ECG and ICG electrodes (1.5-inch disposable silver electrodes; Mindware Technologies, Ltd.) were attached to the participant’s torso and placed under the right collar bone, at the anterior point of the sternum, just under the lower right and left ribs, and on the chest near the apex of the heart. Two more disposable leads were placed on the back of the neck and the lower back. Following placement of the electrodes, signal quality was assessed and, if necessary, adjustments were made to ensure a clear signal. RSA was measured in milliseconds and calculated for each 60-s segment of data collected. Data collection was initiated and processed using Mindware Version 3.1.7.

### Collection and storage of stool samples

Participants were provided with a flushable paper toilet accessory designed to drape from the toilet seat and catch stool prior to exposure to urine or water. Fecal samples were then collected from the paper toilet accessory using sterile plastic applicators and stored in cylindrical plastic collection tubes that contained 2 mL of stabilizing fluid. Stabilizing fluid preserved the sample without freezing or refrigeration for up to 60 days. The stabilizing fluid was a chelating agent which has been shown to effectively preserve fecal samples at ambient temperatures. The stabilizing agent contained Ethyl Alcohol at 10–30% and Sodium Hydroxide at 1–5%. Samples were mailed to the researchers and stored in a freezer at approximately -80 °C by research personnel and remained there until pyrosequencing was conducted.

### Amplicon sequencing: Study 1 only

*(This section was authored and provided with permission by ZymoBIOMICS*® *Service for use in this paper)***.**

The samples were processed and analyzed with the ZymoBIOMICS® Service: Targeted Metagenomic Sequencing (Zymo Research, Irvine, CA). DNA extraction was performed using ZymoBIOMICS®-96 MagBead DNA extraction kit. The DNA samples were prepared for targeted sequencing with the *Quick*-16S™ NGS Library Prep Kit (Zymo Research, Irvine, CA). These primers were custom designed by Zymo Research to provide the best coverage of the 16S gene while maintaining high sensitivity. The primer sets used in this project are marked below. *Quick*-16S™ Primer Set V3-V4 (Zymo Research, Irvine, CA)**.**

The sequencing library was prepared using an innovative library preparation process in which PCR reactions were performed in real-time PCR machines to control cycles and therefore limit PCR chimera formation. The final PCR products were quantified with qPCR fluorescence readings and pooled together based on equal molarity. The final pooled library was cleaned up with the Select-a-Size DNA Clean & Concentrator™ (Zymo Research, Irvine, CA), then quantified with TapeStation® (Agilent Technologies, Santa Clara, CA) and Qubit® (Thermo Fisher Scientific, Waltham, WA).

Control Samples: The ZymoBIOMICS® Microbial Community Standard (Zymo Research, Irvine, CA) was used as a positive control for each DNA extraction, if performed. The ZymoBIOMICS® Microbial Community DNA Standard (Zymo Research, Irvine, CA) was used as a positive control for each targeted library preparation. Negative controls (i.e., blank extraction control, blank library preparation control) were included to assess the level of bioburden carried by the wet-lab process. Sequencing: The final library was sequenced on Illumina® MiSeq™ with a v3 reagent kit (600 cycles) and resulted in an average of 44,832 ± 12,184 reads from the 62 samples. The sequencing was performed with 10% PhiX spike-in. Bioinformatics Analysis: Unique amplicon sequences were inferred from raw reads using the Dada2 pipeline^94^. Chimeric sequences were also removed with the Dada2 pipeline. Taxonomy assignment was performed using Uclust from Qiime v.1.9.1. Taxonomy was assigned with the Zymo Research Database, a 16S database that is internally designed and curated, as reference. Independent taxonomic assignment using the Silva database^95^ was conducted and confirmed identification of common taxa.

Absolute Abundance Quantification*: A quantitative real-time PCR was set up with a standard curve. The standard curve was made with plasmid DNA containing one copy of the 16S gene and one copy of the fungal ITS2 region prepared in tenfold serial dilutions. The primers used were the same as those used in Targeted Library Preparation. The equation generated by the plasmid DNA standard curve was used to calculate the number of gene copies in the reaction for each sample. The PCR input volume (2 µl) was used to calculate the number of gene copies per microliter in each DNA sample. The number of genome copies per microliter DNA sample was calculated by dividing the gene copy number by an assumed number of gene copies per genome. The value used for 16S copies per genome is 4. The value used for ITS copies per genome is 200. The amount of DNA per microliter DNA sample was calculated using an assumed genome size of 4.64 × 10^6^ bp, the genome size of *Escherichia coli*, for 16S samples, or an assumed genome size of 1.20 × 10^7^ bp, the genome size of *Saccharomyces cerevisiae*, for ITS samples. This calculation is shown below:

*Calculated Total DNA* = *Calculated Total Genome Copies* × *Assumed Genome Size (4.64* × *10*^*6*^* bp)* × *Average Molecular Weight of a DNA bp (660 g/mole/bp) ÷ Avogadro’s Number (6.022* × *10*^*23*^*/mole).*

## Method: Study 2

### Participants

The current study recruited mothers of children ages 8 to 12 years old in the Orange and Los Angeles County areas. Research assistants posted advertisements on websites (e.g., Craigslist, public Facebook groups) that parents and children might frequent in the Orange and Los Angeles County areas. Recruitment occurred from March 2018 through August 2019. Once contacted, research staff briefly explained the requirements, purpose of the study (they were told it was a study on child development), screened participants for eligibility, and set up an appointment for the parent–child dyad to participate if qualified. Only six participants reported taking antibiotics within the past month and the remaining participants reported taking them within the past 6 months or longer (i.e., within the last year, 5 years or longer). None of these were excluded. Participants were excluded if they were non-English speakers, diagnosed with autism spectrum disorder (ASD), intellectual disability, or any other mental or physical diagnoses that would inhibit the ability to understand directions or physically perform study tasks. Mothers with a night shift work schedule were also excluded, due to complications in physiological data collection. One hundred mothers completed the main study and 74 completed the microbiome portion of the study (see Supplemental Fig. 2). Mothers and children received a total of $70 in cash and gift cards for completing both parts of the study. Only mothers’ data are included in the current study. The study was approved by the Institutional Review Board at the University of California, Irvine (HS# 2017–3772). We confirm that all experiments were performed in accordance with IRB guidelines and regulations.

### Procedures

The study consisted of two parts: the laboratory visit and the at-home microbiome session. During the laboratory visit, baseline questionnaires were completed, and RSA measures were collected at rest and in reactivity to a stressor directed at the participant’s child while the mother observed the child undergo the stressful task. The at-home microbiome session included online self-reported assessments of stress, health, and diet. The fecal sample was also collected at-home and mailed to the researchers for storage and later sent to another institution to be assayed for microbial composition.

### The laboratory visit

Upon arrival, parents provided informed consent for participation in the in-lab and at-home portions of the study and permission for their children’s participation. Next, mothers completed surveys measuring stress, health, diet, and psychological variables of interest. The dyad commenced the experimental portion of the study, which involved the completion of a laboratory stressor. First, mothers were connected to ECGs – their signals were monitored continuously throughout the study. BioLab 3.3.1 was used for data processing and analysis.

Baseline (resting) RSA was collected for two minutes in a quiet room with the child present. Following baseline, mothers completed the Performance Challenge Task^96–98^ (PCT), a standardized laboratory paradigm used in prior studies to examine dyadic parent–child reactions to children’s stressors. The mother watched her child work independently on a series of geometric puzzles modeled after the Block Design task in the WISC III-R^99^. The task included a demonstration, one practice puzzle, and six unsolvable puzzles (participants were unaware that the puzzles were unsolvable). Mothers watched children as they were given an opportunity to work on the impossible puzzles for seven minutes. To underscore failure on the task, a frown face appeared on partially completed puzzles. Mothers and children were also shown a progress bar at the top of the computer screen showing the percentage of puzzles completed correctly. Maternal RSA was measured for the duration of the seven-minute stress task. After completing the study, both members of the dyad were debriefed and informed that the puzzles were impossible to solve.

### The at-home microbiome session

Upon completion of the in-lab portion of the study, participants were invited to participate in the microbiome portion of the study. Participants also received pre-paid shipping materials to send their samples back. They were instructed not to make any major dietary changes prior to sample collection, and to collect their samples and mail them to the researchers within 3–14 days of participation in the in-lab portion of the study (see Study 1 methods for collection and storage of stool samples).

## Measures

Study 2 used several of the same measures described in Study 1: The PSS^91^, the Holmes-Rahe Life Stress Inventory^52^, the SF-36^92^, and the REAP-S^93^. The procedural description of the collection and storage of stool samples was also the same as in Study 1.

### Demographics and body composition questionnaires

Participants self-reported demographics (race/ethnicity, education, and income). Following the at-home microbiome sample collection, participants were asked by email to self-report height and weight. Participants’ height and weight were used to calculate BMI.

### Cardiovascular

RSA measures were collected using ECG and ICG equipment. Five disposable ECG and ICG electrodes (1.5-inch disposable silver electrodes; Mindware Technologies, Ltd.) were attached to the participant’s torso and placed under the right collar bone, at the anterior point of the sternum, just under the lower right and left ribs, and on the chest near the apex of the heart. Two more disposable leads were placed on the back of the participant on the back of the neck and the lower back. Following placement of the electrodes, signal quality was assessed and, if necessary, adjustments were made to ensure a clear signal. RSA was measured in milliseconds and calculated for each 60-s segment of data collected. Data collection was initiated and processed using BioLab 3.3.1.

### Amplicon sequencing: Study 2 only

*(This section was authored and provided with permission by ZymoBIOMICS*® *Service for use in this paper)*.

The samples were processed and analyzed with the ZymoBIOMICS® Service: Targeted Metagenomic Sequencing. ZymoBIOMICS®-96 MagBead DNA Kit was used to extract DNA. The DNA samples were prepared for targeted sequencing with the Quick-16S™ NGS Library Prep Kit. These primers were custom designed by Zymo Research to provide the best coverage of the 16S gene while maintaining high sensitivity. The primer sets used in this project were the Quick-16S™ Primer Set V3-V4.

Sequencing Analysis: The sequencing library was prepared using an innovative library preparation process in which PCR reactions were performed in real-time PCR machines to control cycles and therefore limit PCR chimera formation. The final PCR products were quantified with qPCR fluorescence readings and pooled together based on equal molarity. The final pooled library was cleaned up with the Select-a-Size DNA Clean & Concentrator™ (Zymo Research, Irvine, CA), then quantified with TapeStation® (Agilent Technologies, Santa Clara, CA) and Qubit® (Thermo Fisher Scientific, Waltham, WA).

Control Samples: The ZymoBIOMICS® Microbial Community Standard was used as a positive control for each DNA extraction. The ZymoBIOMICS® Microbial Community DNA Standard was used as a positive control for each targeted library preparation. Negative controls (i.e., blank extraction control, blank library preparation control) were included to assess the level of bioburden carried by the wet-lab process. Sequencing: The final library was sequenced on Illumina® MiSeq™ with a v3 reagent kit (600 cycles) and resulted in an average of 37,112 ± 9,708 reads from 74 samples. The sequencing was performed with 10% PhiX spike-in. Bioinformatics Analysis: Unique amplicon sequences were inferred from raw reads using the Dada2 pipeline^94^. Chimeric sequences were also removed with the Dada2 pipeline. Taxonomy assignment was performed using Uclust from Qiime v.1.9.1. Taxonomy was assigned with the Zymo Research Database, a 16S database that is internally designed and curated, as reference. Independent taxonomic assignment using the Silva database^95^ was conducted and confirmed identification of major taxa.

Absolute Abundance Quantification*: A quantitative real-time PCR was set up with a standard curve. The standard curve was made with plasmid DNA containing one copy of the 16S gene and one copy of the fungal ITS2 region prepared in tenfold serial dilutions. The primers used were the same as those used in Targeted Library Preparation. The equation generated by the plasmid DNA standard curve was used to calculate the number of gene copies in the reaction for each sample. The PCR input volume (2 µl) was used to calculate the number of gene copies per microliter in each DNA sample. The number of genome copies per microliter DNA sample was calculated by dividing the gene copy number by an assumed number of gene copies per genome. The value used for 16S copies per genome is 4. The value used for ITS copies per genome is 200. The amount of DNA per microliter DNA sample was calculated using an assumed genome size of 4.64 × 106 bp, the genome size of *Escherichia coli,* for 16S samples, or an assumed genome size of 1.20 × 107 bp, the genome size of Saccharomyces cerevisiae, for ITS samples. This calculation is shown below:

*Calculated Total DNA* = *Calculated Total Genome Copies* × *Assumed Genome Size (4.64* × *106 bp)* × *Average Molecular Weight of a DNA bp (660 g/mole/bp) ÷ Avogadro’s Number (6.022* × *1023/mole).*

### Statistical approach

Bacterial relative abundances were derived from QIIME^100^, a bioinformatics tool used to perform microbiome analysis from raw DNA sequences, and assessed using SPSS and/or R. To preserve statistical power, only bacterial genera that were present in at least 10% of the samples were included in primary analyses, resulting in the exclusion of 90 genera and inclusion of 111 genera in Study 1, and the exclusion of 98 genera and inclusion of 85 genera in Study 2. All stress data in both studies are continuous, but tertiles were calculated to create a 2/3^rd^ group for all high stress groups and a 1/3^rd^ group for all low stress groups as done in similar research assessing associations between stress and microbial composition^101^. Stress group assignment was needed to calculate differences in beta diversity and tertiles were chosen to provide finer distinction between high and low stress groups^102^. Permutational analysis of variance analyses (PERMANOVA) of Bray–Curtis dissimilarities using 999 permutations was used to quantify variation in genera (centroids and dispersion) between the groups. Specifically, beta diversity was calculated using the adonis2 function in Vegan package 2.6–4 to determine Bray–Curtis distance matrices. PERMANOVA^103^ significance testing for compositional data was then performed. Nonmetric multidimensional scaling (NMDS) ordination was obtained from the ‘metaMDS’ function in Vegan to plot beta diversity.

Perceived stress, stressful life events, and RSA/physiological stress tertile groups were used for all analyses assessing alpha and beta diversity; when PERMANOVAs were statistically significant, post-hoc analyses were conducted to determine which microbes differentiated stress groups from each other. The stress group sizes in the current studies were comparable and often larger than groups found in extant human stress-microbiome literature^101,104,105^. [In both studies, power analyses were conducted in G*power software^106^ using the “Means: Difference between two independent samples means (two groups)” as the statistical test and the “post hoc” option for the type of power analysis. Calculations indicated that Study 1 was powered to detect a large effect size (0.80) with the power set at 0.81, and Study 2 was powered to detect a large effect size (0.80) with the power set at 0.87]. PERMANOVAs that revealed significant differences between low, mid, and high stress groups were followed with subsequent PERMANOVAs assessing each pairwise comparison to parse group differences. Additionally, significant PERMANOVAs were probed to discover which microbes differentiated stress groups using analysis of composition of microbiomes with bias correction (ANCOM-BC), a microbiome specific statistical methodology that accounts for underlying compositional structure in the microbiome. ANCOM-BC corrects for multiple comparisons, normalizes zero-inflated abundances, calculates linear regressions, provides effect size (log fold change; lfc), and identifies structural zeros when groups are completely missing or nearly completely missing a particular taxon^107^. The identification of structural zeros is one qualitative assessment used in microbiome work to identify the presence and absence of certain taxa^108,109^. As with all previously mentioned analyses, ANCOM-BC analyses included all genera present in at least 10% of the samples. These analyses were conducted in an exploratory manner; however, the ANCOM-BC approach corrects for multiple comparisons and only adjusted *p*-values are reported.

### Correlation and regression analyses for select genera

Zero-order correlations among perceived stress, stressful life events, RSA baseline, RSA reactivity, and bacterial genera were conducted to test whether these variables were significantly intercorrelated as continuous variables. These analyses were performed to investigate associations between levels of stress within each stressor type in all 62 participants in Study 1 and all 74 participants in Study 2 and select genera. Further, genus selection was based on extant literature linking certain taxa to stress to reduce multiple comparisons. Measures of select genera were transformed using centered log-ratio^110^; however, relative abundance values in significant models (prior to transformation) are reported in the results section.

Multiple comparison procedures were used to control for the false discovery rate. False discovery rates (q-values) in genome and metagenome (microbiome) research vary and include *q*-values such as 0.15^111^. In the current study, we considered a *p*-value < 0.05 and a *q*-value < 0.15 significant as has been done in past similar work^111^. To probe significant findings further, linear regression models (e.g., hierarchical regressions) were conducted using continuous variables as used in similar work^112,113^. Specifically, we used hierarchical regressions and adjusted for sex (in Study 1 only), diet, overall health, BMI, and age. Covariates were chosen based on prior research suggesting that sex^114^, diet^115^, overall health, BMI^116,117^, and age^118^ influence gut microbial profiles. This group of covariates will now be referred to as physical covariates. RSA baseline was adjusted for in RSA reactivity analyses for both studies by including it as a covariate. We include two steps per model: step 1 includes variables for which we adjust for covariates and step 2 includes the predictor of interest. The adjusted R^2^ and *p*-values of step 1 of each model and the R^2^ change, unstandardized beta coefficient, standard error, and *p*-values of step 2 are reported for each significant model. Correlation matrices for variables in statistically significant models in both studies are reported in Supplemental Tables 6 and 11.

### Analysis of gut microbial functional pathways

Phylogenetic Investigation of Communities by Reconstruction of Unobserved States^119^ is a tool used to predict functional pathways of metagenomes in microbial ecosystems based on identified 16S rRNA gene sequences in samples. Low abundance genes were removed at < 0.1% of the maximum count. The pathway abundance counts were log2 transformed for analysis. Contrast analysis within the framework of the general linear model was applied to test for differences in the pathway abundances by stress domain group membership. We also applied the general linear model to test for associations between the pathway abundances and levels of stress in each stress domain controlling for age, BMI, sex, overall health, and diet. We provide effect size measures, Cohen’s *d* for mean differences, and the standardized beta for association analysis. We applied a false discovery rate of 5% and reported the adjusted *p*-value, *q*. We considered *q* < 0.25 as the reporting threshold and emphasize effect size estimation over significance testing in this relatively small sample.

### Overview of Study 1 and Study 2 microbiota composition

The relative abundance of microbial families across all samples in both studies are depicted in Supplemental Figs. 3 and 4. Note that the top 10 most abundant taxa depicted in Supplemental Figs. 3 and 4 are similar between both studies. Specifically, the only differences between the most common bacteria displayed in each study were the inclusion of *Verrucomicrobiaceae* in Study 1 and the inclusion of *Rikenellaceae* in Study 2. Further, the top 10 most abundant taxa in with research showing that the prevalence of both *Bifidobacterium*^120^ both studies complement research attempting to define a universal core of taxa shared by humans. When compared to data from around the world, namely, El Salvador, Madagascar, Peru, China, Japan, and the United States, the most abundant bacteria in both studies corresponded to the universal core of taxa proposed by Piquer-Estaban and colleagues^121^ (2021), with the exception of *Bifidobacteriaceae, Erysipelotrichaceae, Peptostreptococcaceae,* and *Verrucomicrobiaceae*, bacterial families that include genera and species that tend to be more prevalent in the gut microbiome of those who live in industrialized countries^122,123^*.* Additionally, means and standard deviations of relative abundance values for all select bacteria in both studies, namely, *Lactobacillus, Akkermansia, Bifidobacterium, Streptococcus, Escherichia/Shigella*, and *Clostridium,* prior to log centered ratio transformations, are reported in Supplemental Table 12. Notably, the prevalence of some of the select genera is not well-established in healthy samples and varies by individual. However, the values displayed in Supplemental Table 12 coincide and *Clostridium*^121^ in the human gut typically exceeds 90% in healthy adults. Finally, it is important to note that some microbes were indistinguishable from each other during taxonomic assignment. In such instances, microbes were classified together (*i.e., Escherichia/Shigella* and *Lachnoclostridium/Roseburia*).

## Supplementary Information

Supplementary Information 1.

Supplementary Information 2.

## Data Availability

Study 1: The sequencing data that support the findings of this study are openly available on the Sequence Read Archive at https://www.ncbi.nlm.nih.gov/sra/PRJNA1164305, under the BioProject ID, PRJNA1164305. Study 2: The sequencing data that support the findings of this study are openly available on the Sequence Read Archive at https://www.ncbi.nlm.nih.gov/sra/PRJNA1164400, under the BioProject ID, PRJNA1164400.
