# Supplementary material for: Biological, environmental, and psychological stress and the human gut microbiome in healthy adults
Source: Sci Rep. 2025 Jan 2;15:362. doi: 10.1038/s41598-024-77473-9 (PMC11695967; doi:10.1038/s41598-024-77473-9)
Supplement: Supplementary file 1 — Supplementary Information 1. [file 41598_2024_77473_MOESM1_ESM.pptx]

## Slide 1
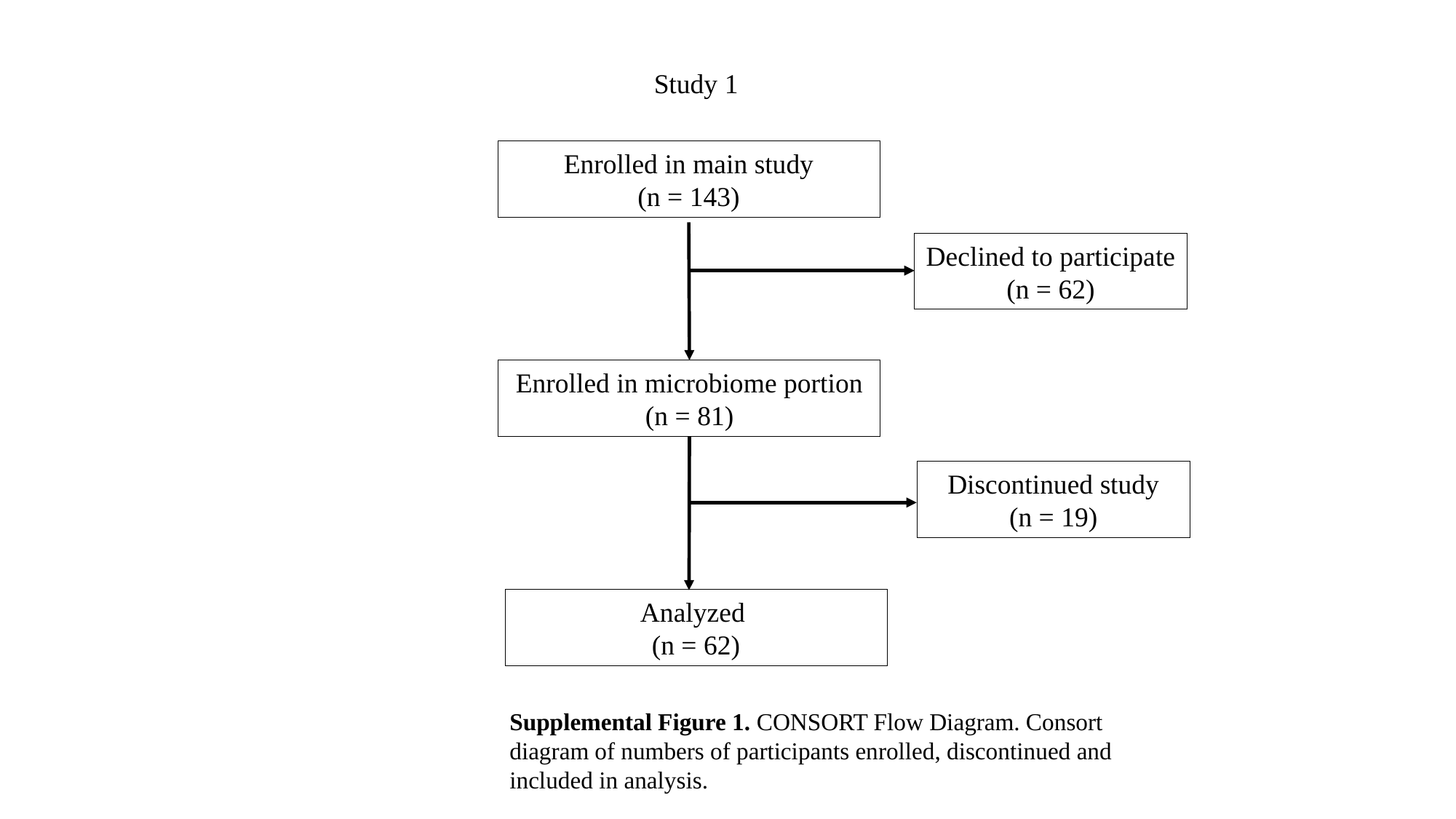

Study 1
Enrolled in main study
(n = 143)
Declined to participate
(n = 62)
Enrolled in microbiome portion (n = 81)
Discontinued study
(n = 19)
Analyzed
(n = 62)
Supplemental Figure 1. CONSORT Flow Diagram. Consort diagram of numbers of participants enrolled, discontinued and included in analysis.

## Slide 2
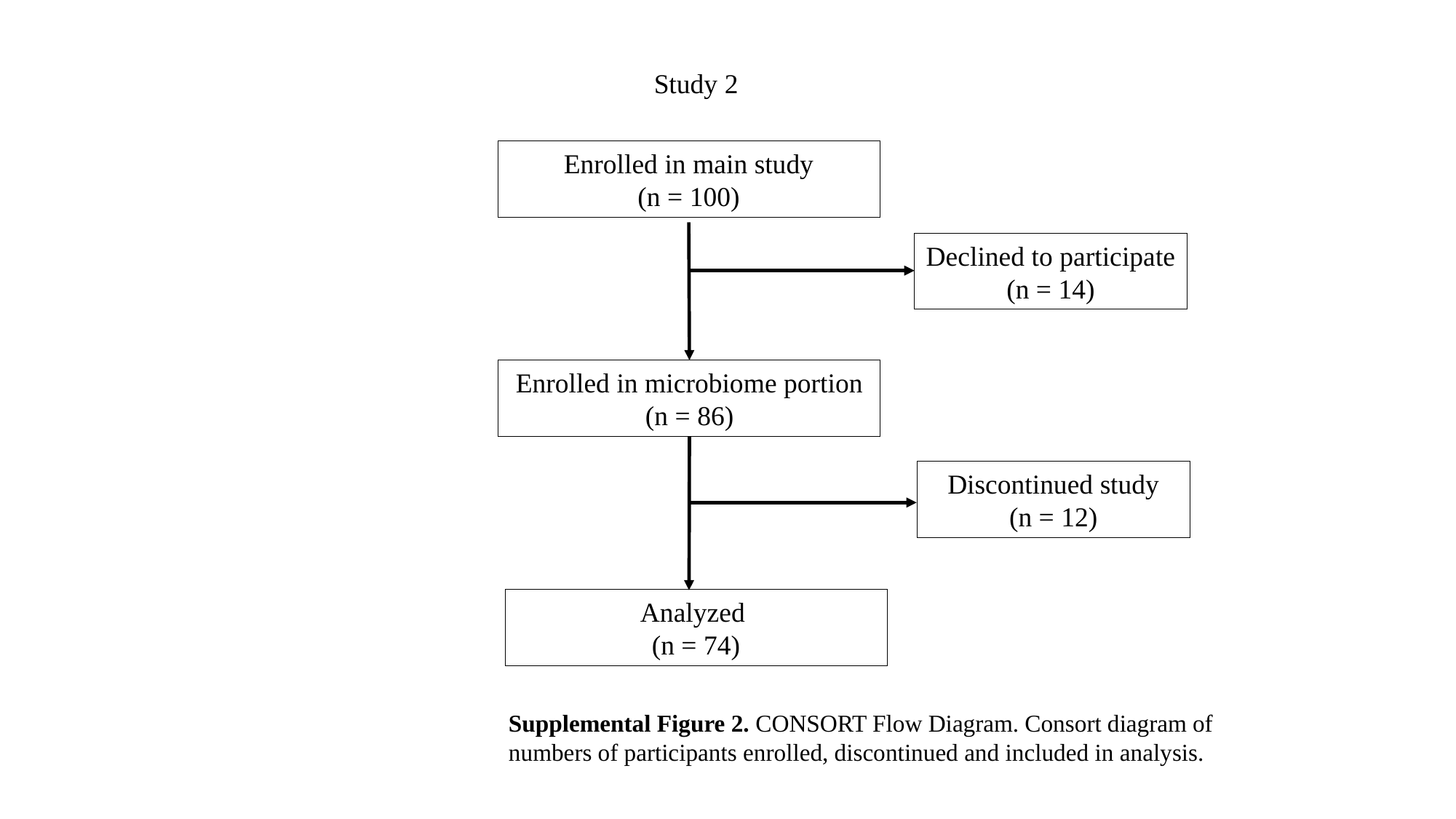

Study 2
Enrolled in main study
(n = 100)
Declined to participate
(n = 14)
Enrolled in microbiome portion (n = 86)
Discontinued study
(n = 12)
Analyzed
(n = 74)
Supplemental Figure 2. CONSORT Flow Diagram. Consort diagram of numbers of participants enrolled, discontinued and included in analysis.

## Slide 3
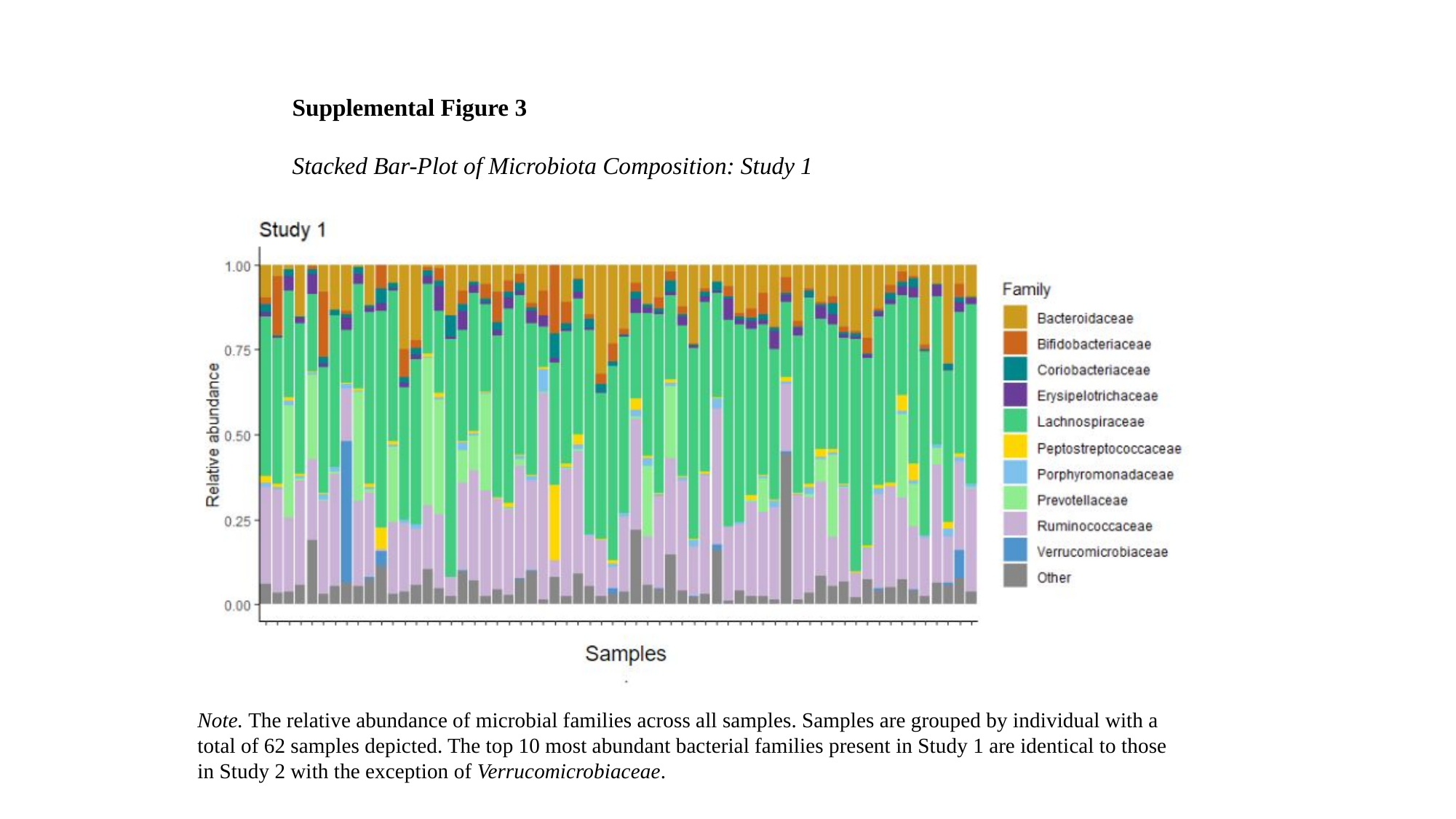

Supplemental Figure 3
Stacked Bar-Plot of Microbiota Composition: Study 1
Note. The relative abundance of microbial families across all samples. Samples are grouped by individual with a total of 62 samples depicted. The top 10 most abundant bacterial families present in Study 1 are identical to those in Study 2 with the exception of Verrucomicrobiaceae.

## Slide 4
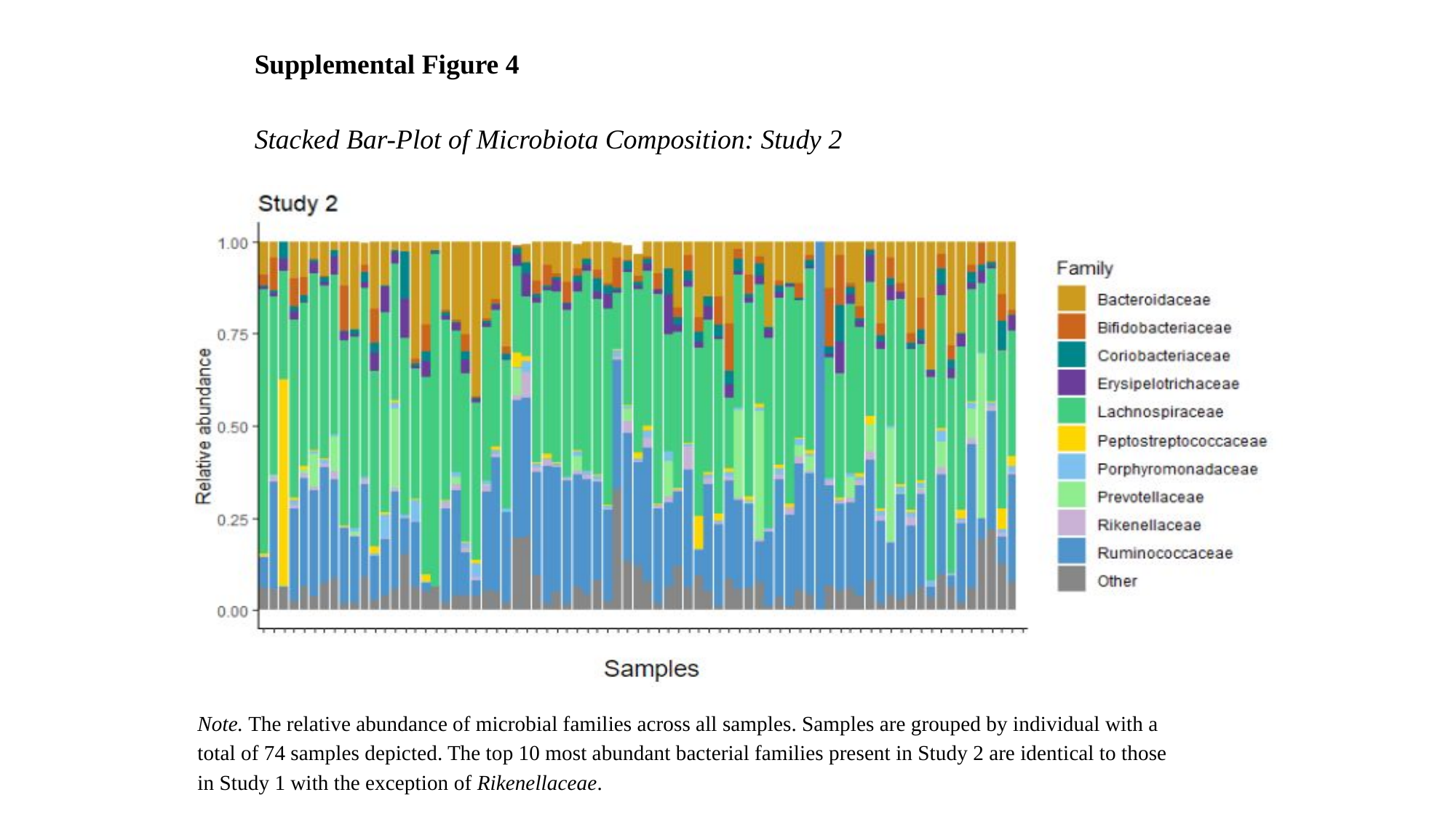

Supplemental Figure 4
Stacked Bar-Plot of Microbiota Composition: Study 2
Note. The relative abundance of microbial families across all samples. Samples are grouped by individual with a total of 74 samples depicted. The top 10 most abundant bacterial families present in Study 2 are identical to those in Study 1 with the exception of Rikenellaceae.
