# Supplementary material for: Biological, environmental, and psychological stress and the human gut microbiome in healthy adults
Source: Sci Rep. 2025 Jan 2;15:362. doi: 10.1038/s41598-024-77473-9 (PMC11695967; doi:10.1038/s41598-024-77473-9)
Supplement: Supplementary file 2 — Supplementary Information 2. [file 41598_2024_77473_MOESM2_ESM.docx]

| **Supplemental Table 1**. Statistically Non-Significant Differences in Beta Diversity between Stress Groups in Study 1 | | | | | |
| --- | --- | --- | --- | --- | --- |
| **Factor** | **df** | **SS** | **Pseudo F** | **R^2^** | ***p*** |
|  |  |  |  |  |  |
| Low-Mid Stressful Life Events Groups | 1 | 0.115 | 1.168 | 0.028 | 0.278 |
|  |  |  |  |  |  |
| Residual | 40 | 3.930 |  | 0.971 |  |
|  |  |  |  |  |  |
| Total | 41 | 4.045 |  | 1.000 |  |
|  |  |  |  |  |  |
|  |  |  |  |  |  |
| Mid-High Stressful Life Events Groups | 1 | 0.212 | 1.762 | 0.042 | 0.059 |
|  |  |  |  |  |  |
| Residual | 40 | 4.819 |  | 0.958 |  |
|  |  |  |  |  |  |
| Total | 41 | 5.031 |  | 1.000 |  |
|  |  |  |  |  |  |
|  |  |  |  |  |  |
| Low, Mid, High Perceived Stress Groups | 2 | 0.167 | 0.727 | 0.024 | 0.817 |
|  |  |  |  |  |  |
| Residual | 59 | 6.76 |  | 0.976 |  |
|  |  |  |  |  |  |
| Total | 61 | 6.93 |  | 1.000 |  |
|  |  |  |  |  |  |
|  |  |  |  |  |  |
| df, degrees of freedom; SS, sum of squares; Results of permutational analysis of variance | | | | | |

| **Supplemental Table 2.** The Detection of Structural Zeros in Stressful Life Events Groups in Study 1 | | | | | | | | | |
| --- | --- | --- | --- | --- | --- | --- | --- | --- | --- |
| **Taxon** | **Low Stressful Life Events** | | | **Mid Stressful Life Events** | | | **High Stressful Life Events** | | |
|  | **Structural**  **Zero** | **Sample**  **Frequency** | **Sample**  **Prevalence** | **Structural**  **Zero** | **Sample**  **Frequency** | **Sample**  **Prevalence** | **Structural**  **Zero** | **Sample**  **Frequency** | **Sample Prevalence** |
| *Acidaminococcus* | false | 7/20 | 0.650 | true | 3/22 | .136 | true | 3/20 | 0.150 |
| *Megasphaera* | false | 5/20 | 0.250 | true | 3/22 | .136 | true | 3/20 | 0.150 |
| *Veillonella* | false | 4/20 | 0.200 | true | 2/22 | .090 | true | 2/20 | 0.100 |
| *Faecalibacterium/Subdoligranulum* | false | 5/20 | 0.250 | false | 7/22 | .318 | true | 3/20 | 0.150 |
| *Intestinimonas* | false | 6/20 | 0.300 | false | 5/22 | .227 | true | 3/20 | 0.150 |
| *Slackia* | true | 3/20 | 0.150 | false | 5/22 | .227 | false | 8/20 | 0.400 |
| *Anaerofustis* | true | 0/20 | 0.00 | false | 4/22 | .182 | false | 6/20 | 0.300 |
| *Anaerofilum* | true | 2/20 | 0.100 | false | 5/22 | .227 | false | 4/20 | 0.200 |
| *Coprobacter* | true | 2/20 | 0.100 | false | 6/22 | .272 | true | 0/20 | 0.000 |
| *Enterococcus* | true | 2/20 | 0.100 | false | 4/22 | .182 | true | 2/20 | 0.100 |
| *Eubacterium/Roseburia* | true | 2/20 | 0.100 | false | 6/22 | .272 | true | 1/20 | 0.050 |
| *Pseudobutyrivibrio* | true | 2/20 | 0.100 | false | 7/22 | .318 | true | 2/20 | 0.100 |
| *Gelria* | true | 2/20 | 0.100 | false | 4/22 | .182 | true | 2/20 | 0.100 |
| *Stoquefichus* | true | 2/20 | 0.100 | false | 5/22 | .227 | true | 2/20 | 0.100 |
| *Oxalobacter* | true | 3/20 | 0.150 | false | 5/22 | .227 | true | 2/20 | 0.100 |
| *Klebsiella* | true | 1/20 | 0.050 | false | 5/22 | .227 | true | 2/20 | 0.100 |
| *Enterorhabdus* | true | 3/20 | 0.150 | true | 2/22 | .090 | false | 4/20 | 0.200 |
| *Olsenella* | true | 2/20 | 0.100 | true | 1/22 | .045 | false | 4/20 | 0.200 |
| *Eubacterium* | true | 3/20 | 0.150 | true | 1/22 | .045 | false | 4/20 | 0.200 |
| *Howardella* | true | 1/20 | 0.050 | true | 2/22 | .090 | false | 4/20 | 0.200 |
| *Lactonifactor* | true | 2/20 | 0.100 | true | 3/22 | .136 | false | 4/20 | 0.200 |
| *Holdemanella* | true | 3/20 | 0.150 | true | 2/22 | .090 | false | 6/20 | 0.300 |
| *Coprobacillus* | true | 2/20 | 0.100 | true | 1/22 | .045 | false | 4/20 | 0.200 |
| *Note.* Taxa displayed are reported due to ANCOM-BCs identification of structural zeros in either the high or low  stressful life events groups for Study 1. | | | | | | | | | |

| **Supplemental Table 3**. Statistically Non-Significant Differences in Alpha Diversity between Stress Groups in Study 1 | | | | | |
| --- | --- | --- | --- | --- | --- |
| **Variable** | **df** | **SS** | **Mean Sq** | **F** | ***p*** |
|  |  |  |  |  |  |
| Stressful Life Events Groups | 2 | 0.059 | 0.030 | 0.369 | 0.693 |
| Residuals | 59 | 4.724 | 0.080 |  |  |
|  |  |  |  |  |  |
| RSA Stress Reactivity Groups | 2 | 0.024 | 0.012 | 0.150 | 0.861 |
| Residuals | 59 | 4.759 | 0.081 |  |  |
|  |  |  |  |  |  |
|  |  |  |  |  |  |
| df, degrees of freedom; SS, sum of squares; Low, mid, and high stress groups were included in these analyses of variance | | | | | |

| **Supplemental Table 4**. Study 1- Correlations between Six Select Genus and Continuous Stress Domain | | | | | | | | |
| --- | --- | --- | --- | --- | --- | --- | --- | --- |
|  |  |  |  |  |  |  |  |  |
| **Variables** | **1** | **2** | **3** | **4** | **5** | **6** | **7** | **8** |
|  |  |  |  |  |  |  |  |  |
| 1. Stressful Life Events | -- |  |  |  |  |  |  |  |
|  |  |  |  |  |  |  |  |  |
| 2. Perceived Stress | 0.157 | -- |  |  |  |  |  |  |
|  |  |  |  |  |  |  |  |  |
| 3. RSA Stress Reactivity | 0.104 | 0.172 | -- |  |  |  |  |  |
|  |  |  |  |  |  |  |  |  |
| *4. Lactobacillus* | -0.016 | 0.117 | 0.057 | -- |  |  |  |  |
|  |  |  |  |  |  |  |  |  |
| *5. Akkermansia* | 0.035 | 0.000 | -0.201 | 0.081 | -- |  |  |  |
|  |  |  |  |  |  |  |  |  |
| *6. Bifidobacterium* | -0.030 | 0.037 | -0.193 | 0.089 | -0.060 | -- |  |  |
|  |  |  |  |  |  |  |  |  |
| *7. Streptococcus* | -0.127 | -0.182 | 0.140 | -0.138 | 0.038 | 0.092 | -- |  |
|  |  |  |  |  |  |  |  |  |
| *8. Escherichia/Shigella* | -0.038 | -0.005 | -.203 | 0.111 | 0.141 | 0.155 | .183 | -- |
|  |  |  |  |  |  |  |  |  |
| *9. Clostridium* | 0.047 | 0.190 | -0.369** | 0.115 | 0.016 | 0.149 | -.181 | 0.278* |
|  |  |  |  |  |  |  |  |  |
|  |  |  |  |  |  |  |  |  |
| *Note*. * *p* < .05., ** *p* < .01. | | | | | | | | |

| **Supplemental Table 5.** The Detection of Structural Zeros in RSA Reactivity Groups in Study 1 | | | |
| --- | --- | --- | --- |
| **Taxon** | **Low RSA** | **Mid RSA** | **High RSA** |
| *Slackia* | true | false | false |
| *Blautia/Marvinbryantia* | true | false | false |
| *Faecalibacterium/Subdoligranulum* | true | false | false |
| *Acidaminococcus* | true | false | false |
| *Megasphaera* | true | false | false |
| *Thalassospira* | true | false | false |
| *Pseudobutyrivibrio* | true | true | false |
| *Holdemanella* | true | true | false |
| *Coprobacillus* | false | true | true |
| *Enterorhabdus* | true | false | true |
| *Allisonella* | true | false | true |
| *Veillonella* | true | false | true |
| *Klebsiella* | false | true | true |
| *Cloacibacillus* | false | true | true |
| *Coprobacter* | false | true | true |
| *Enterococcus* | false | true | true |
| *Anaerofustis* | false | true | true |
| *Eubacterium* | false | true | true |
| *Eubacterium_1* | false | true | true |
| *Eubacterium/Roseburia* | false | true | true |
| *Lactonifactor* | false | true | true |
| *Anaerofilum* | false | true | true |
| *Gelria* | false | true | true |
| *Stoquefichus* | false | true | false |
| *Escherichia/Shigella* | false | true | false |
| *Denitrobacterium* | false | false | true |
| *Oxalobacter* | false | false | true |
| *Note.* Taxa displayed in this table are reported due to ANCOM-BCs identification of structural zeros in either the high or low RSA reactivity group in Study 1. | | | |

| **Supplemental Table 6.** Correlation Matrix for Key Variables in Study 1 | | | | | | | | | |
| --- | --- | --- | --- | --- | --- | --- | --- | --- | --- |
| **Variables** | **1** | **2** | **3** | **4** | **5** | **6** | **7** | **8** | **9** |
| 1. Age in Years | -- |  |  |  |  |  |  |  |  |
| 1. Sex | .125 | -- |  |  |  |  |  |  |  |
| 1. Body Mass Index | .030 | -.154 | -- |  |  |  |  |  |  |
| 1. General Health | -.351** | .129 | .032 | -- |  |  |  |  |  |
| 1. Meat Consumption | -.017 | .105 | -.368** | -.206 | -- |  |  |  |  |
| 1. Vegetable/Fruit Consumption | .213 | .154 | -.123 | -.124 | .175 | -- |  |  |  |
| 1. Grain Consumption | .230 | .160 | -.110 | -.069 | .088 | .315* | -- |  |  |
| 1. RSA Baseline | -.187 | -.103 | -.066 | .021 | .089 | -.023 | -.213 | -- |  |
| 1. RSA Stress Reactivity | -.407** | -.073 | -.149 | .180 | .059 | -.030 | -.140 | .593** | -- |
| 1. *Clostridium* | -.381** | -.028 | -.014 | -.045 | -.101 | -.078 | -.072 | -.098 | -.369** |
| *Note*: Sex Coding; 1 = men, 2 = women  * *p* < .05. ** *p* < .01. *** *p* < .001  Includes correlations between variables in significant models. | | | | | | | | | |

| **Supplemental Table 7.** The Detection of Structural Zeros in Stressful Life Events Groups in Study 2 | | | | | | | | | |
| --- | --- | --- | --- | --- | --- | --- | --- | --- | --- |
| **Taxon** | **Low Stressful Life Events** | | | **Mid Stressful Life Events** | | | **High Stressful Life Events** | | |
|  | **Structural**  **Zero** | **Sample**  **Frequency** | **Sample**  **Prevalence** | **Structural**  **Zero** | **Sample**  **Frequency** | **Sample**  **Prevalence** | **Structural**  **Zero** | **Sample**  **Frequency** | **Sample**  **Prevalence** |
| *Terrisporobacter* | false | 4/24 | .166 | true | 2/25 | .080 | true | 3/25 | .120 |
| *Enterorhabdus* | false | 4/24 | .166 | true | 2/25 | .080 | true | 3/25 | .120 |
| *Denitrobacterium* | false | 7/24 | .292 | true | 2/25 | .080 | true | 3/25 | .120 |
| *Shuttleworthia* | false | 4/24 | .166 | false | 5/25 | .200 | true | 2/25 | .080 |
| *Lactobacillus* | false | 5/24 | .208 | false | 5/25 | .200 | true | 2/25 | .080 |
| *Note.* Taxa displayed in Table 4 are reported due to ANCOM-BCs identification of structural zeros in either the high or low stressful life events groups for Study 2. | | | | | | | | | |

| **Supplemental Table 8.** Statistically Non-Significant Differences in Alpha Diversity between Stress Groups in Study 2 | | | | | |
| --- | --- | --- | --- | --- | --- |
|  | | | | | |
| **Variable** | **df** | **Sum Sq** | **Mean Sq** | **F** | ***p*** |
|  |  |  |  |  |  |
| Stressful Life Events Groups | 2 | 0.135 | 0.067 | 0.825 | 0.443 |
| Residuals | 71 | 5.811 | 0.082 |  |  |
|  |  |  |  |  |  |
|  |  |  |  |  |  |
| Perceived Stress Groups | 2 | 0.090 | 0.044 | 0.544 | 0.583 |
| Residuals | 71 | 5.856 | 0.082 |  |  |
|  |  |  |  |  |  |
|  |  |  |  |  |  |
| RSA Stress Reactivity Groups | 2 | 0.076 | 0.038 | 0.458 | 0.634 |
| Residuals | 71 | 5.870 | 0.083 |  |  |
|  |  |  |  |  |  |
|  |  |  |  |  |  |
| df, degrees of freedom; Low, mid, and high stress groups were included in ANOVA these analyses. | | | | | |

| **Supplemental Table 9**. Statistically Non-Significant Differences in Beta Diversity between Stress Groups in Study 2 | | | | | |
| --- | --- | --- | --- | --- | --- |
|  |  |  |  |  |  |
| **Factor** | **df** | **SS** | **Pseudo F** | **R^2^** | ***p*** |
|  |  |  |  |  |  |
|  |  |  |  |  |  |
| Low-Mid Stressful Life Events Groups | 1 | 0.134 | 1.338 | 0.028 | 0.205 |
|  |  |  |  |  |  |
| Residual | 47 | 4.719 |  | 0.972 |  |
|  |  |  |  |  |  |
| Total | 48 | 4.853 |  | 1.00 |  |
|  |  |  |  |  |  |
|  |  |  |  |  |  |
| Low-High Perceived Stress Groups | 1 | 0.133 | 1.300 | 0.026 | 0.203 |
|  |  |  |  |  |  |
| Residual | 49 | 5.011 |  | 0.974 |  |
|  |  |  |  |  |  |
| Total | 50 | 5.144 |  | 1.00 |  |
|  |  |  |  |  |  |
|  |  |  |  |  |  |
| Low-Mid Perceived Stress Groups | 1 | 0.091 | 0.841 | 0.018 | 0.597 |
|  |  |  |  |  |  |
| Residual | 47 | 5.114 |  | 0.982 |  |
|  |  |  |  |  |  |
| Total | 48 | 5.210 |  | 1.00 |  |
|  |  |  |  |  |  |
|  |  |  |  |  |  |
| Mid- High Perceived Stress Groups | 1 | 0.056 | 0.469 | 0.010 | 0.940 |
|  |  |  |  |  |  |
| Residual | 46 | 5.480 |  | 0.990 |  |
|  |  |  |  |  |  |
| Total | 47 | 5.537 |  | 1.00 |  |
|  |  |  |  |  |  |
|  |  |  |  |  |  |
| Low-Mid-High RSA Stress Reactivity Groups | 2 | 0.121 | 0.545 | 0.015 | 0.765 |
|  |  |  |  |  |  |
| Residual | 71 | 7.870 |  | 0.985 |  |
|  |  |  |  |  |  |
| Total | 73 | 7.992 |  | 1.00 |  |
|  |  |  |  |  |  |
|  |  |  |  |  |  |
| df, degrees of freedom; SS, sum of squares; Results of permutational analysis of variance | | | | | |

| **Supplemental Table 10.** Study 2- Correlations between Six Select Genus and Continuous Stress Domain | | | | | | | | |
| --- | --- | --- | --- | --- | --- | --- | --- | --- |
| **Variables** | **1** | **2** | **3** | **4** | **5** | **6** | **7** | **8** |
|  |  |  |  |  |  |  |  |  |
| 1. Stressful Life Events | -- |  |  |  |  |  |  |  |
|  |  |  |  |  |  |  |  |  |
| 2. Perceived Stress | 0.090 | -- |  |  |  |  |  |  |
|  |  |  |  |  |  |  |  |  |
| 3. RSA Stress Reactivity | -0.066 | -.089 | -- |  |  |  |  |  |
|  |  |  |  |  |  |  |  |  |
| *4. Lactobacillus* | 0.124 | 0.133 | 0.300* | -- |  |  |  |  |
|  |  |  |  |  |  |  |  |  |
| *5. Akkermansia* | -0.039 | -0.041 | -0.010 | 0.148 | -- |  |  |  |
|  |  |  |  |  |  |  |  |  |
| *6. Bifidobacterium* | 0.048 | -0.045 | -0.054 | 0.156 | 0.100 | -- |  |  |
|  |  |  |  |  |  |  |  |  |
| *7. Streptococcus* | -0.028 | -0.132 | 0.103 | -0.144 | -0.075 | -0.125 | -- |  |
|  |  |  |  |  |  |  |  |  |
| *8. Escherichia/Shigella* | 0.115 | 0.270* | 0.120 | 0.113 | -0.084 | -0.127 | -0.171 | -- |
|  |  |  |  |  |  |  |  |  |
| *9. Clostridium* | 0.030 | -0.204 | -0.016 | -0.013 | 0.099 | -0.203 | -0.162 | .073 |
|  |  |  |  |  |  |  |  |  |
| *Note*. * *p* < .05. | | | | | | | | |

| **Supplemental Table 11***.* Correlation Matrix for Key Variables in Study 2 *(n = 74)* | | | | | | | | | | |
| --- | --- | --- | --- | --- | --- | --- | --- | --- | --- | --- |
| **Variables** | **1** | **2** | **3** | **4** | **5** | **6** | **7** | **8** | **9** | **10** |
| 1. Age in Years | -- |  |  |  |  |  |  |  |  |  |
| 1. Body Mass Index | -.129 | -- |  |  |  |  |  |  |  |  |
| 1. General Health | -.011 | -.238* | -- |  |  |  |  |  |  |  |
| 1. Meat Consumption | .172 | -.252* | .151 | -- |  |  |  |  |  |  |
| 1. Vegetable/Fruit Consumption | .051 | -.089 | .013 | .077 | -- |  |  |  |  |  |
| 1. Grain Consumption | .067 | .116 | -.073 | -.066 | .313** | -- |  |  |  |  |
| 1. RSA Baseline | -.305** | -.171 | .131 | .021 | -.013 | -.057 | -- |  |  |  |
| 1. RSA Stress Reactivity | -.263* | -.084 | .078 | -.027 | -.118 | -.003 | .807** | -- |  |  |
| 1. Perceived Stress | .050 | .049 | -.365** | .107 | -.002 | -.004 | -.055 | -.089 | -- |  |
| 1. *Lactobacillus* | .004 | -.295* | -.196 | -.160 | -.110 | -.151 | .193 | .269* | .142 | -- |
| 1. *Escherichia/Shigella* | .155 | -.005 | -.112 | .014 | -.053 | -.113 | .158 | .100 | .264* | .113 |
| *Note*. * *p* < .05. ** *p* < .01. *** *p* < .001  Includes correlations between variables in significant models. | | | | | | | | | | |

| **Supplemental Table 12.** Relative Abundance and Prevalence of Select Bacteria in Study 1 and Study 2 | | | | |
| --- | --- | --- | --- | --- |
| ***Select Bacteria*** | **Study 1 (Total *N* = 62)**  ***M(SD)*** | **Study 2 (Total *N* = 74)**  ***M(SD)*** | **Study 1**  **Prevalence** | **Study 2**  **Prevalence** |
| *Lactobacillus* | .140(.689) | .071(.368) | 33.87 | 16.22 |
| *Akkermansia* | 1.003(5.36) | .270(.620) | 48.39 | 43.24 |
| *Bifidobacterium* | 3.010(4.277) | 3.126(3.64) | 85.48 | 82.43 |
| *Streptococcus* | .389(.705) | .962(2.477) | 93.55 | 90.54 |
| *Escherichia/Shigella* | .601(4.13) | .147(.371) | 33.87 | 35.14 |
| *Clostridium* | .489(.353) | .273(.619) | 98.39 | 48.65 |
| *Note.* Values of relative abundances prior to centered log transformation and prevalence of select genus in percentage form are reported in this table. | | | | |

| **Supplemental Table 13.** Descriptive Statistics of Key Variables by Sex for Study 1 (*n* = 62) | | | | |
| --- | --- | --- | --- | --- |
| *Measures* | Total *N* = 62  *M(SD)* | Males *n* = 17 | Females *n* = 45 | *Sex differences*  *t* |
| Age in Years | 37.601(11.696) | 35.244(10.574) | 38.491(12.084) | -1.043 |
| Body Mass Index | 25.528(6.420) | 27.119(6.097) | 24.927(6.502) | 1.241 |
| General Health | 33.681(13.168) | 30.951(12.664) | 34.713(13.346) | -1.028 |
| Meat Consumption | 2.322(.5132) | 2.235(.471) | 2.355(.529) | -.863 |
| Vegetable/Fruit Consumption | 2.274(.630) | 2.117(.740) | 2.334(.582) | -1.086 |
| Grain Consumption | 2.371(.751) | 2.176(.808) | 2.444(.724) | -1.196 |
| RSA Baseline | 5.725(1.173) | 5.920(1.103) | 5.651(1.203) | .833 |
| RSA Stress Reactivity | 5.778(1.283) | 5.930(1.410) | 5.721(1.244) | .537 |
| *Clostridium* | -.478(1.657) | -.404(1.817) | -.506(1.614) | .203 |
| *Note.* Includes descriptive statistics for variables in significant models. No sex differences were found. Taxa abundance data displayed in this Table were adjusted for compositionally with centered log-ratio transformation. Relative abundance values for participants (*N* = 62) in Study 2, prior to transformation are as follows: *Clostridium* (*M* = .488, *SD* = 1.352)  Abbreviations: Respiratory Sinus Arrhythmia (RSA). | | | | |

| **Supplemental Table 14.** Descriptive Statistics of Key Variables: Study 2 | | |
| --- | --- | --- |
| *Measures* | Total *N* = 74  *M SD* | |
| Age in Years | 41.592 | 6.349 |
| Body Mass Index | 25.649 | 6.551 |
| General Health | 73.413 | 18.283 |
| Meat Consumption | 2.299 | .533 |
| Vegetable/Fruit Consumption | 2.464 | .573 |
| Grain Consumption | 2.291 | .804 |
| RSA Baseline | 5.960 | 1.326 |
| RSA Stress Reactivity | 5.649 | 1.123 |
| Perceived Stress | 14.381 | 6.465 |
| *Lactobacillus* | -.262 | .862 |
| *Escherichia/Shigella* | -.372 | .818 |
| *Note.* Includes descriptive statistics for variables in significant models. Taxa abundance data displayed in this Table were adjusted for compositionally with centered log-ratio transformation. Relative abundance values, prior to transformation are as follows: *Lactobacillus* (*M* = .070, *SD* = .368). *Escherichia /Shigella* (*M* = .147, *SD* = .371) | | |
